# Hierarchical Network Enabled Flexible Textile Pressure Sensor with Ultrabroad Response Range and High‐Temperature Resistance

**DOI:** 10.1002/advs.202105738

**Published:** 2022-03-14

**Authors:** Meiling Jia, Chenghan Yi, Yankun Han, Lei Wang, Xin Li, Guoliang Xu, Ke He, Nianci Li, Yuxin Hou, Zhongguo Wang, Yuanhao Zhu, Yuanao Zhang, Mingzhu Hu, Ran Sun, Peifei Tong, Jiawei Yang, Yougen Hu, Zhixun Wang, Weimin Li, Wenjie Li, Lei Wei, Chunlei Yang, Ming Chen

**Affiliations:** ^1^ Center for Photonics Information and Energy Materials Shenzhen Institute of Advanced Technology Chinese Academy of Sciences Shenzhen 518055 P. R. China; ^2^ Department of Nano Science and Technology Institute University of Science and Technology of China Suzhou 215123 P. R. China; ^3^ School of Computer and Control Engineering University of Chinese Academy of Sciences Beijing 100049 P. R. China; ^4^ Shenzhen Institutes of Advanced Electronic Materials Shenzhen Institutes of Advanced Technology Chinese Academy of Sciences Shenzhen 518055 P. R. China; ^5^ School of Electrical and Electronic Engineering Nanyang Technological University 50 Nanyang Avenue Singapore 639798 Singapore

**Keywords:** high pressure, high temperature, linearity range, polyimide fabric, pressure sensors, sensing range

## Abstract

Thin, lightweight, and flexible textile pressure sensors with the ability to detect the full range of faint pressure (<100 Pa), low pressure (≈KPa) and high pressure (≈MPa) are in significant demand to meet the requirements for applications in daily activities and more meaningfully in some harsh environments, such as high temperature and high pressure. However, it is still a significant challenge to fulfill these requirements simultaneously in a single pressure sensor. Herein, a high‐performance pressure sensor enabled by polyimide fiber fabric with functionalized carbon‐nanotube (PI/FCNT) is obtained via a facile electrophoretic deposition (EPD) approach. High‐density FCNT is evenly wrapped and chemically bonded to the fiber surface during the EPD process, forming a conductive hierarchical fiber/FCNT matrix. Benefiting from the large compressible region of PI fiber fabric, abundant yet firm contacting points and high elastic modulus of both PI and CNT, the proposed pressure sensor can be customized and modulated to achieve both an ultra‐broad sensing range, long‐term stability and high‐temperature resistance. Thanks to these merits, the proposed pressure sensor could monitor the human physiological information, detect tiny and extremely high pressure, can be integrated into an intelligent mechanical hand to detect the contact force under high‐temperature.

## Graphical abstract

High‐performance pressure sensor enabled by polyimide fiber fabric with functionalized carbon‐nanotube (PI/FCNT) via a facile electrophoretic deposition approach is reported. Benefiting from the large compressible region of PI fiber fabric, abundant yet firm contacting points and high elastic modulus of both PI and CNT, the proposed pressure sensor achieves both an ultra‐broad sensing range, long‐term stability and high‐temperature resistance.

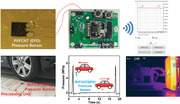

## 1 Introduction

Thin, flexible, and wearable pressure sensors have a great application perspective in electronic skins, healthcare monitors, soft robotics, artificial intelligence, etc.^[^ ^1^, ^2^, ^3^, ^4^, ^5^, ^6^, ^7^, ^8^, ^9^, ^10^ ^]^ To date, flexible pressure sensors are mainly divided into four types: capacitive, piezoelectric, triboelectric, and piezoresistive.^[^ ^11^, ^12^, ^13^, ^14^, ^15^, ^16^, ^17^, ^18^, ^19^, ^20^ ^]^ Among the proposed flexible pressure sensors, piezoresistive sensors are widely used owing to their low manufacturing cost, ease of fabrication as well as easy readout mechanism. Flexible piezoresistive pressure sensors are generally comprised of three parts: flexible matrix material (such as the frequently used polydimethylsiloxane (PDMS), polyurethane (PU)), conductive materials (such as Au, Ag, graphene, CNT), and electrode materials.

With the change of practical applications and customer needs, in addition to fulfilling the requirements of daily life (such as wearable healthcare monitoring at normal pressure and room temperature), there is an increasing demand for wearable pressure sensors that can be used in harsh environments, such as high temperatures and/or high pressures. For instance, real‐time physiological monitoring of firefighters during the firefight action is critical to assess the level of danger and make a real‐time and life‐saving decision to protect firefighters.^[^ ^21^, ^22^, ^23^ ^]^ Besides, the real‐time detection of prestresses (≈tens of MPa) within the composite sheath of high‐speed permanent magnet motor rotor under high temperature (>300 °C) is vital for the whole motor system, which requires a flexible yet thin pressure sensor with the applicability in both high‐temperature and high‐pressure condition.^[^ ^24^, ^25^, ^26^ ^]^ Thus, there is an urgent need to achieve a single pressure sensor that can detect the full range of faint pressure (<100 Pa), low‐pressure (in the range of KPa, such as human motions), high pressure (in the range of MPa, for example, safety monitoring of road, rail, bridge, and tunnel) and can be used in harsh high‐temperature and high‐pressure environments. However, it is still a significant challenge.

**Figure 1:**
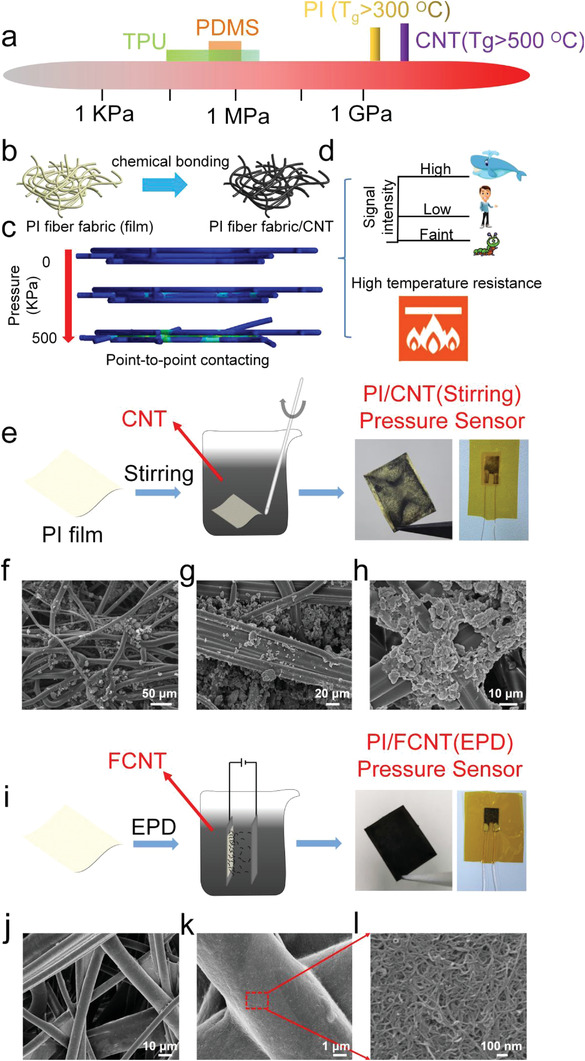
Materials selection, structure optimization, sensing mechanism, and the fabricated PI/CNT (stirring), PI/FCNT(EPD) pressure sensors. a) Materials selection: schematic illustration of Young's modulus of frequently used flexible matrix materials and conductive materials for the pressure sensors. (b) Structure optimization: PI fiber‐based fabrics and CNT is adopted as flexible matrix materials and conductive materials, respectively. c) Sensing mechanism: contacting points variation of the PI fiber fabrics/CNT under external pressure from 0 to 500 KPa. d) Schematic illustration of the response of the proposed PI fiber fabrics/CNT pressure sensor to faint, low, and high pressure, and such proposed pressure sensor possesses the merit of high‐temperature resistance. e) Schematic illustration of the fabrication process of PI/CNT (stirring) fabric, optical images of the PI/CNT(Stirring) fabric, and the final encapsulated PI/CNT(Stirring) pressure sensor. f–h) Top‐view SEM images of the PI/CNT(Stirring) fabric. i) Schematic illustration of the fabrication process of PI/FCNT(EPD) fabric, optical images of the PI/FCNT(EPD) fabric, and the final encapsulated PI/FCNT(EPD) pressure sensor. j–l) Top‐view SEM images of PI/FCNT(EPD) fabric.

Generally, high modulus of the sensor material is a necessary precondition to the broad sensing range and large linearity. A typical method to improve the linearity is to create nanosized or microsized patterns on elastic polymer. For example, several PDMS geometrical features, including micropyramids, microhemispheres, and microsemicylinders, are demonstrated to be an effective method to improve the linearity.^[^ ^27^, ^28^, ^29^, ^30^ ^]^ In our previous work,^[^ ^31^, ^32^, ^33^, ^34^ ^]^ we also show that the contacting modes play a substantial effect on the sensing performance and demonstrate that the point‐to‐point contacting mode is the optimum strategy. However, these elastic polymers are commonly subject to the disadvantage of low elastic modulus (10 KPa to 10 MPa) and low glass transition temperature, which reveals that the sensor response will saturate at low‐pressure levels (namely narrow linearity range and sensing range), and the sensor is not suitable for the high‐temperature environments. Besides, the fabrication of these micro/nano‐structured elastic polymer/conductive layers often requires complex fabrication processes, such as UV lithography, ICP/RIE etching, E‐beam evaporation/magnetron sputtering, etc. To address these issues, we adopt polyimide (PI) as the flexible matrix material, because PI possesses the merits of higher modulus and high‐temperature‐resistant properties compared to other elastic polymers^[^ ^35^, ^36^ ^]^ (**Figure** **1**a). However, high modulus usually leads to increased stiffness. Namely, the deformation of PI under the external pressure is not large, which may result in low sensitivity and quick signal saturation. Therefore, PI film with a wide compressible region is an essential prerequisite for large linearity and wide sensing range, and the appropriate design of contacting modes is crucial in order to offer large and consistent resistance change during the compressible region. Chen et al. reported a wearable pressure sensor based on PI/CNT composite aerogels through the freeze‐drying and thermal imidization process, showing a linear range of 5 KPa and a sensing range of 61 KPa. The sensing mechanism can be attributed to the contact of the adjacent cell walls (surface contacts) upon external compression.^[^ ^37^ ^]^ Jeong et al. proposed a flexible pressure sensor based on tip flattened microdome shaped PI film and CNT via conventional lithography, RIE etching, and lift‐off process. The sensor presents a sensing range of 3 MPa, a maximum sensitivity of 5.66 × 10^−3^ KPa^−1^ at 50 KPa and a minimum sensitivity of 0.23 × 10^−3^ KPa^−1^ at 3 MPa.^[^ ^38^ ^]^ However, these PI/CNT pressure sensors cannot fulfill the above‐mentioned requirements.

Herein, a facile and robust electrophoretic deposition (EPD) process is used to deposit functionalized carbon nanotubes (FCNTs) on PI fiber‐based fabrics under an electric field. The fabricated PI fiber fabrics possess a large compressible region (from “loose” PI fiber fabric to “compact” PI fiber fabric), which is very beneficial to the sensor's linear range and sensing range. Within the PI fiber‐based fabrics, plenty of micro‐ and nanohierarchical pores built by the multilayer PI microfiber networks endow the matrix material with a large specific surface (more contacting points), high structural stability, and high flexibility (Figure 1b, Figures S1 and S2, Supporting Information). After the EPD process, the FCNTs are chemically bonded to the PI fiber surface, forming a conductive hierarchical network (Figure 1b). Thanks to the large compressible region of PI fiber fabric, abundant yet firm contacting points, point‐to‐point contacting mode (Figure 1c), and high elastic modulus of both PI and CNT, we demonstrate a thin (≈0.33 mm), lightweight (86.5 mg cm_‐_^3^), flexible and wearable pressure sensor that is able to detect pressures from below 100 Pa all the way up to 45 MPa, which corresponds to the range from faint pressure (airflow, the weight of an aluminum particle) to low pressure (pulse beat, joint movement) to very high pressure (the weight of an automobile). Furthermore, thanks to the intrinsic temperature resistance of both PI and CNT, the proposed sensor also exhibit a sensitivity of 38.66 and 2.85 MPa^−1^ in the wide linear ranges of 0–36 KPa and 36 KPa–6.88 MPa at 200 °C and can work at higher temperatures above 300 °C, indicating its high applicability in harsh high‐temperature and high‐pressure environments (Figure 1d).

## 2 Results and Discussion

In order to obtain a uniform and conductive hierarchical network based on PI fiber fabric/CNT structure, four sets of pressure sensors, namely PI/CNT(stirring), PI/FCNT(stirring), PI/CNT(EPD), and PI/FCNT(EPD), are designed and fabricated. First, a conventional mechanical stirring method was used to incorporate CNT into the PI fiber fabric (Figure 1e, PI/CNT (stirring)). As shown in the right session of Figure 1e, only part of the PI fiber fabric turns black while other regions remain unchanged. The underlying reason for this nonuniformity is due to the agglomeration behavior of CNT during the stirring process,^[^ ^39^ ^]^ as confirmed in Figure 1f–h. Furthermore, these agglomerated CNT tend to be gathered in the gaps of the PI fibers rather than the PI surface (Figure 1f–h, Figure S3a, Supporting Information). To improve the uniformity, a highly acidic and thermal oxidation process was used as the initial step to modify the CNT. This process not only was beneficial to the wettability and dispersibility of the CNT, but also made the CNT functionalized with negatively charged carboxyl group.^[^ ^40^, ^41^ ^]^ An EPD process^[^ ^42^ ^]^ was then adopted to deposit FCNT on PI fiber fabric (Figure 1i). Under an electric field, these FCNTs migrated to the PI fiber fabric (anode) and were evenly wrapped to the fiber surface (Figure S4a, Supporting Information), as evidenced by the optical and SEM images of PI/CNT(EPD) (Figure 1j–l). It is worth noting that benefiting from the high‐porous PI fiber fabric structure, these FCNTs would migrate to each fiber and be uniformly coated onto the fiber surface, even for the fibers adhered to the anode (Figure S4b–d, Supporting Information). Figures S3b and S5a (Supporting Information) represent the doping mechanism and the morphology of PI/FCNT(stirring) and PI/CNT(EPD), respectively. Compared to PI/CNT(Stirring), FCNT is relatively evenly distributed on the PI fiber surface for PI/FCNT (stirring), however, the coverage degree is still far below that of PI/FCNT(EPD). The least effective doping method is the EPD process with CNT. It is observed that only a thin layer of CNTs was adhered to one surface (away from the anode) of the PI fiber fabric (Figure S5b, Supporting Information). This is well understood because CNT cannot move directionally to the PI fibers under an applied electric field. In general, the optical and SEM morphology images show that the best strategy for obtaining a uniform and conductive network is through adopting the modified CNT with the EPD process. It is also worth noting that a “self‐assembled” method can also be adopted to develop the uniform and conductive PI/CNT network, however, the process may be complex and time‐consuming.^[^ ^43^ ^]^ Conceivably, compared to the other three kinds of pressure sensors, the abundant contacting points within the conductive hierarchical network will be very beneficial to enhance the sensitivity, sensing range, and reliability of the PI/FCNT(EPD) pressure sensor.

**Figure 2:**
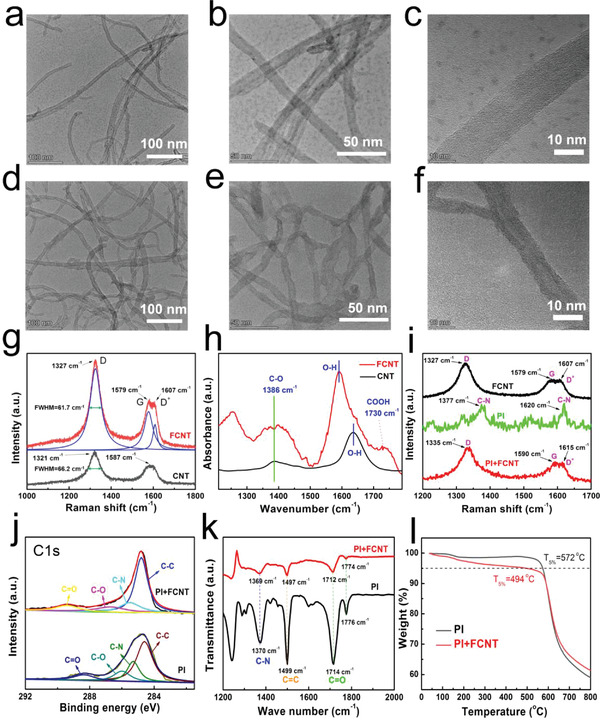
Characterization of CNT, FCNT, PI, and PI/FCNT(EPD). a–c) TEM images of CNTs. d–f) TEM images of FCNTs. g) Representative Raman spectra of CNT and FCNT. h) FT‐IR spectra of CNT and FCNT. i) Representative Raman spectra of PI, FCNT, and PI/FCNT(EPD). j) XPS survey (C1s) spectra of PI and PI/FCNT(EPD). k) FT‐IR spectra of PI and PI/FCNT (EPD). l) Thermogravimetric analysis curves of PI and PI/FCNT (EPD).

The above results indicate that the modified CNT plays an important role in the proposed PI/FCNT(EPD) pressure sensor. To further probe into the intrinsic reason, we explored the interaction between PI fiber and FCNT, physical properties, and structure difference between FCNT and CNT by transmission electron microscopy (TEM), Raman spectroscopy, Fourier transform infrared (FTIR) spectroscopy, and X‐ray photoelectron spectroscopy (XPS). **Figure** **2**a–c and d–f are the TEM images of CNT and FCNT, respectively. The tube diameter is decreased from ≈16 nm for CNT to ≈8 nm for FCNT, which is helpful to improve the dispersion.^[^ ^44^, ^45^ ^]^ This partly accounts for the better uniformity for PI/FCNT (stirring) compared with PI/CNT (stirring). Raman spectra of CNT and FCNT are shown in Figure 2g. Two characteristic peaks, located at 1321 cm^−1^ (D band) and 1587 cm^−1^ (G band), are observed for CNT. After modification, it shows a similar Raman spectrum (D band and G band) but with a more distinguishable D+ band (1607 cm^−1^). Furthermore, FCNT possesses a narrower full‐width at half maximum (FWHM) of 61.7 cm^−1^ compared with that of as‐received CNT (66.2 cm^−1^). This is because the modification process improves the structural order and purity of the CNT.^[^ ^46^ ^]^ Here, the D and D+ bands represent a double‐resonance Raman mode due to the amorphous carbon, disorder, defects, or ion intercalation between the graphitic walls. The G band is due to the tangential in‐plane stretching vibrations of the carbon‐carbon bonds within the graphene sheets.

FTIR spectroscopy analysis of the FCNT provides the direct evidence of the successful modification with CNT (Figure 2h), showing the shifted O–H absorption peak compared with CNT and characteristic band at 1730 cm^−1^ which is due to C═O stretching vibration within the carboxyl and carbonyl functional groups.^[^ ^46^ ^]^ Note that this characteristic peak is not observed in CNT. In addition, Other notable peaks such as 1386 cm^−1^ observed in both CNT and FCNT is ascribed to the C—O stretching vibration.

The existence of strong chemical interactions between FCNT and PI can be deduced from the Raman spectra, XPS, and FTIR analysis, as shown in Figure 2i–k. As for the Raman spectra shown in Figure 2i, two characteristic peaks of PI at 1377 and 1620 cm^−1^ are observed, corresponding to the stretch vibration of C–N within the imide ring and the aromatic imide ring vibrations of the dianhydride portion (green line). As excepted, PI/FCNT(EPD) Raman spectra (red line) yielded a Raman spectrum containing the D‐, G‐, and D+ bands of FCNT, which moved to a high‐frequency position compared to that of FCNT. In general, the Raman spectra of PI/FCNT(EPD) overlap and broaden the characteristic peaks of PI and FCNT, which is mainly attributed to the charge transfer between PI and FCNT. The average of the charge density between them causes the peak overlap and broadening.^[^ ^37^, ^47^, ^48^ ^]^ The XPS spectra in Figure S6 (Supporting Information) show the characteristic peaks of both PI and PI/FCNT(EPD) centered at C1s (285.2 eV), N1s(400 eV), and O1s(532 eV). Compared with PI, the PI/FCNT(EPD) demonstrates an increment of C1s and O1s peak intensity and decrement of N1s peak intensity, which is owing to the existence of FCNT. Figure 2j represents the C1s spectrum, the relative contributions of the C—C, C—N, C—O, C═O are 54.11%, 21.26%, 14.38%, 10.24% for PI, and 59.79%, 16.82%, 12.62%, 10.77% for PI/FCNT (EPD). There is a decreased intensity of the C—N and C—O for PI/FCNT(EPD) compared to that of the PI, which shows the existence of chemical interactions between FCNT and PI.^[^ ^40^ ^]^ FTIR analysis of PI and PI/FCNT (EPD) was further performed, as displayed in Figure 2k. Several notable absorption peaks located at 1370, 1499, 1714, and 1776 cm^−1^ are clearly observed for PI, caused by C—N, C═C stretching vibration, C═O symmetric, and asymmetric stretching, respectively. For PI/FCNT, these absorption peaks shifted to a low wavenumber direction, and the intensity is significantly reduced due to the strong interaction between PI and FCNT. These strong chemical interactions come from the created strong hydrogen bonds between C═O and C═N of imide ring and carboxyl groups of FCNT^,[^ ^37^, ^47^, ^48^ ^]^ To demonstrate that the bonding happens only with the FCNT, we have also characterized the FTIR for the PI/CNT(Stirring) film. The result is shown in Figure S7 (Supporting Information). We did not find the absorption peak movement for PI/CNT(Stirring) film, and the intensity for absorption peaks is comparable to PI film. In a word, the obtained uniform and conductive PI/FCNT (EPD) film is attributed to the directional migration of FCNT during the EPD process as well as the formed strong hydrogen bonds between PI and FCNT. Figure 2l shows the thermogravimetric analysis curves of PI and PI/FCNT (EPD). At low temperature, the weight loss for PI/FCNT(EPD) is higher than that of PI. We believe the reason mainly includes two aspects: 1) the thermal conductivity of PI film (≈0.1 W m^‐1^ K^‐1^) is much lower than that of CNT (3000 W m^‐1^ K^‐1^). During the thermogravimetric test (heating rate: 5 °C min^‐1^), under the same environment temperature, the real‐applied temperature for PI film is lower than that of PI/FCNT(EPD) film due to the poor thermal conductivity of PI film, and 2) there might be a little structure degradation of the PI/FCNT composite material during the heating process. It is worth noting that although the thermal stability of the PI/FCNT(EPD) film is decreased, its T_5%_ (the temperature at 5% weight loss, T_5%_) still reaches 494 °C, revealing its excellent thermal stability.

**Figure 3:**
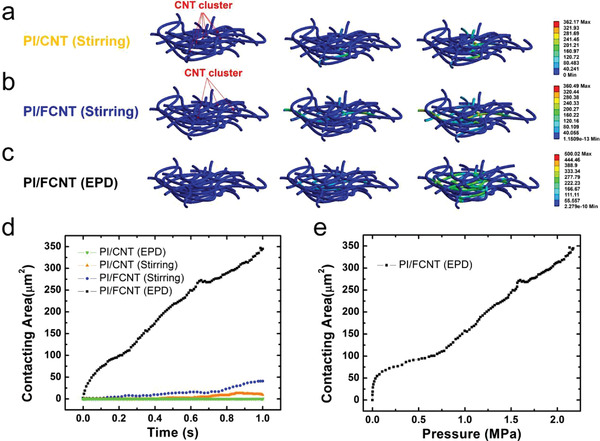
Finite element analysis of proposed pressure sensors. a–c) Microscopic deformation process for the model with PI/CNT(stirring), PI/FCNT(stirring), and PI/FCNT(EPD), respectively. d) Contacting area as a function of time for the proposed four sets of pressure sensors. e) Contacting area versus Pressure curve for PI/FCNT(EPD) pressure sensor.

Next, we consider the sensing performance of the proposed four sets of pressure sensors. To better understand the sensing mechanism for the PI fiber/CNT architectures and compare their sensing performance, Creo/Engineering software is adopted to build the PI fiber/CNT model and finite element modeling (FEM) to analyze the dynamic working process of the pressure sensors under external pressure. A detailed explanation of the FEM simulation can be found in Figures S8–S10 (Supporting Information). Figure S11a–c (Supporting Information) shows the established models for PI/CNT(stirring), PI/FCNT(stirring), and PI/FCNT(EPD), respectively. Note that the PI/CNT(EPD) pressure sensor is not taken into consideration, because there is only a thin layer of CNT adhered to one surface of the PI fiber fabric (Figure S5b, Supporting Information), and the conductive network is not formed. According to the SEM results shown in Figure 1, Figures S3 and S4 (Supporting Information), the established conductive contact mode for PI/CNT(stirring), PI/FCNT(stirring), and PI/FCNT(EPD) is CNT cluster to CNT cluster, CNT cluster to PI/FCNT fibers, PI/FCNT fibers to PI/FCNT fibers (Figure S11a–c, Supporting Information), respectively. Dynamic microscopic deformation processes for these models are displayed in **Figure** **3**a–c and Figures S12–S14 (Supporting Information). Figure 3d shows the contacting area as a function of time for the four sets of pressure sensors. As the time increases from 0 to 1 s, the increment speed of the number contacting for PI/FCNT(EPD) is far above that of the other three types of pressure sensors, indicating that the PI/FCNT(EPD) pressure sensor possesses the highest sensitivity. Besides, the contacting area gradually increases without saturation as the external pressure exceeding 2 MPa, revealing a broad working range for the proposed PI/FCNT(EPD) pressure sensor. The theoretical results presented in Figure 3d,e agree well with the experimental results as detailed below.

**Figure 4:**
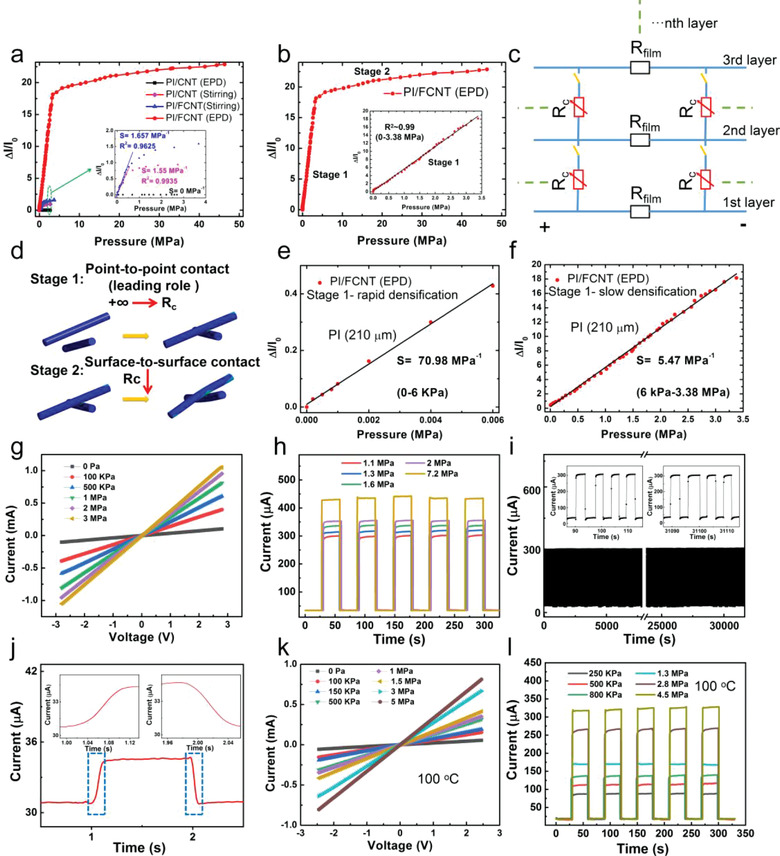
Pressure sensing performance of the fabricated pressure sensors. a) Relative current change versus the pressure applied to the PI/CNT(EPD), PI/CNT(stirring), PI/FCNT(stirring), and PI/FCNT(EPD) pressure sensor. b) Relative current change versus the pressure applied to the PI/FCNT(EPD) pressure sensor. Inset: Plots of current variation versus pressures up to 3.38 MPa and the corresponding sensitivity. c) Equivalent circuit model of the PI/FCNT(EPD) piezoresistive pressure sensor. d) Schematic illustration for the contacting mode between the PI/FCNT (EPD) fibers during the compression process. e,f) Relative current change versus the pressure applied to PI/FCNT(EPD) pressure sensor during the rapid and slow densification region, respectively. g) *I*–*V* curves of the PI/FCNT(EPD) sensor device under different applied pressures. h) Multiple cycles of pressure response under different pressures ranging from 1.1 to 7.2 MPa. i) The durability test for the PI/FCNT(EPD) pressure sensor. j) Response and relaxation time of the PI/FCNT(EPD) pressure sensor. k) *I*–*V* curves with different loading pressure ranging from 0 Pa to 5 MPa at 100 °C. l) The relative current variation of the PI/FCNT(EPD) pressure sensor under repeated pressures ranging from 250 KPa to 4.5 MPa at 100 °C.

To experimentally evaluate the performance of the proposed pressure sensors, we set up an intelligent data‐acquisition device containing a universal electric signal analyzer Keithley 2400 and a computer‐controlled dynamic positioning system. The effects of the CNT morphology and doping technique on the sensitivity of the proposed pressure sensors were studied by measuring the output current change as a function of applied external pressure, as shown in **Figure** **4**a,b. The size of the pressure sensors is 5 mm (length) × 5 mm (width) × 0.33 mm (thickness of the encapsulated sensor). Note that the thickness of the PI fiber fabric is 210 µm, and all the characterization and application shown in the paper are based on this type of PI fiber fabric. As expected, the PI/FCNT(EPD) pressure sensor exhibits higher current change under the same external pressure (namely, higher sensitivity) and broader sensing range than that of other sets of pressure sensors, showing an excellent agreement with the theoretical prediction (Figure 3). PI/FCNT(EPD) pressure sensor exhibits wide linearity and an exceptionally broad sensing range up to 45 MPa. In addition, the fabricated PI/FCNT(EPD) pressure sensors exhibit reliable pressure responses with good uniformity, as shown in Figure S15 (Supporting Information) (3 PI/FCNT (EPD) pressure sensor). The sensing behavior of the PI/FCNT(EPD) pressure sensors can be divided into two stages (Figure 4b). To explain this, an illustration of the equivalent circuit to show the resistance changes under different pressures is shown in Figure 4c,d. In the low‐pressure region (0–3.38 MPa), the PI fiber fabric experienced the densification process, namely, from “loose” PI fiber fabric to “compact” PI fiber fabric. In this region, the point‐to‐point contact working mechanism played the leading role in the resistance change, and the pressure sensor possessed a relatively high sensitivity because of a large amount of fibers contact, leading to the result that the resistance changed abruptly: from infinite (+∞) to *R*_c_. In the high‐pressure region (3.38–45 MPa), the PI/FCNT(EPD) was almost densified. In this region, the resistance change was mainly dependent on the deformation of the PI/FCNT fibers, namely, the “surface‐to‐surface” contact played the primary role in the sensitivity.

We attribute this wide linearity and ultrabroad response range to the large yet appropriate compressible region of PI fiber fabric, abundant yet firm contacting sites (high‐density FCNT is evenly wrapped and chemically bonded to the PI fibers via EPD process), point‐to‐point contacting mode within the hierarchical conductive network, and high elastic modulus of both PI and CNT. The result of wide linearity and broad sensing range is, first of all, determined by the compressible region of the PI film. To demonstrate this, two other types of PI fiber fabrics with thicknesses of 50 and 100 µm are designed and fabricated (Figure S16, Supporting Information). As shown from the compressive stress–strain curve (Figure S17, Supporting Information), the compressible region enlarges with the increase of the PI fiber fabric thickness. We fabricated pressure sensors based on these three types of PI fiber fabrics. The *P*–*I* results are shown in Figure S18 (Supporting Information), demonstrating that the linearity of the pressure sensor is proportional to the compressible region of the PI fiber fabric. In other words, a large compressible region for the PI fiber fabric (210 µm) contributes to the wide linearity. It needs to mention that for the PI/FCNT (EPD) (PI fiber fabric: 210 µm) pressure sensor, there is a very short high sensitivity region (0–6 KPa, corresponding to 0–0.15 N), which may be due to the “rapid densification” of the PI fiber fabric during the initial compressive process (Figure 4e,f). The “rapid densification” region is originated from the relatively low effective modulus of the porous PI fiber fabric. However, for the PI fiber fabrics with the thickness of 50 and 100 µm, the “rapid densification” region is not observed. This is due to the following two reasons: 1) the “rapid densification” region of the thin PI fiber fabric is shorter than that of the thicker PI fiber fabric (Figure S16, Supporting Information); 2) this short “rapid densification” region is further decreased due to the initial imposed pressure during the packaging process. In order to verify the above analysis, we further fabricated the pressure sensor with thicker PI fiber fabric by stacking two PI fiber fabric (210 µm). As excepted, the “rapid densification” working region of the pressure sensor becomes larger (Figure S19, Supporting Information). In other words, the whole linearity is decreased. As a result, PI/FCNT (EPD) pressure sensor based on the PI fiber fabric with the thickness of 210 µm is the optimal selection, which combines the merits of thin, lightweight, flexible, wide linearity, and ultra‐broad sensing range. Besides, as mentioned above, PI film with a wide compressible region is an essential prerequisite for realizing high linearity, however, this is not a sufficient condition. Another important factor is the point‐to‐point contacting mode, which offer large and consistent resistance change during the “loose” PI fiber fabric to “compact” PI fiber fabric region.

To achieve the broad response range as well as large linearity, besides the optimization of PI fiber thickness (Figures S18 and S19, Supporting Information), we also optimized the electric resistance of PI/FCNT(EPD). In the experiments, it is found that the EPD time plays a vital role on the sensing performance (electrical resistance) of the PI/FCNT(EPD) pressure sensor, as shown in Figure S20 (Supporting Information). Several features can be found from the experimental results: 1) The initial electrical resistance decreases (*R*_0_) as the EPD time increases, and this is well understood because more conductive FCNT moved to the PI fibers, resulting in a low *R*_film_ (Figure 4c). 2) When the EPD process time is 20 min, the PI/FCNT(EPD) shows the lowest performance (including sensitivity, linear and sensing range). This is because the PI fibers are only partially covered by the FCNT. As the pressure increases to ≈1.6 MPa, the amount of conductive contacting points (point‐to‐point contact) tends to saturate. 3) When the EPD process time is 3 h, the initial resistance reaches 3.74 kΩ (*R*_0_ = 1 V/267 µA = 3.74 kΩ). This low initial resistance is mainly due to the low resistance of PI film (*R*_film_). Although the current could reach ≈1845 µA under the applied pressure of 22 MPa, the current change (Δ*I*/*I*_0_) is not comparable to that of PI/FCNT(EPD) with 1 h EPD process. This is because the relatively low basic resistance (*R*_film_) (or high initial current *I*_0_), the effect of the change of *R*_c_ is reduced. In other words, although the *R*_c_ continues to decrease as the pressure increases, its effect on the change of total resistance is weakened. More importantly, if the EPD time further increases, it is found that the PI/FCNT(EPD) pressure sensor is not stable under high pressure. We believe the main reason is that more FCNTs are overlapped on the FCNTs rather than chemically bonded onto the PI fiber surface. The bonding force between the thick FCNTs is not strong, and they tend to fall off under high pressure. To sum up, PI/FCNT(EPD) with 1 h EPD could benefit to high sensitivity, broad sensing range and high stability due to 1) relative low initial current (*I*_0_); 2) the PI fiber surface is uniformly covered by FCNT, and the point‐to‐point contacting mode plays the decisive role in the current change; and 3) the PI fiber surface is covered by an appropriate amount of FCNT, where the chemical bonding between the FCNT and PI guarantees the robustness of the sensor.

Our proposed PI/FCNT(EPD) pressure sensor not only can work at high pressure but also can operate at high temperatures. Figure S21 (Supporting Information) are the infrared (IR) thermal imaging images of the PI/FCNT(EPD) pressure sensor placed on a heat source, respectively. It can be seen that even if the heating temperature exceeds 300 °C, the morphology and the temperature distribution of the PI/FCNT(EPD) pressure sensor almost remains the same during a heating period of 5 min. The sensing performance of the PI/FCNT(EPD) pressure sensor at 200 °C is also characterized (see Experimental section), as depicted in Figure S22 (Supporting Information). Note that before the testing, the PI/FCNT (EPD) film was baked at 200 °C for 48 h for the aging process. The proposed sensor device exhibits stable *P*–*I* curves (Figure S23, Supporting Information), and still possesses a sensitivity of 38.66 and 2.85 MPa^−1^ in the wide linear ranges of 0–36 KPa and 36 KPa–6.88 MPa. Overall, these high‐temperature experimental results demonstrated the flame retardancy and good heat stability of the proposed PI/FCNT pressure sensors, enabling their potential to be used in harsh high‐temperature conditions.

Besides the sensitivity and sensing range, another electric characteristic of the PI/FCNT(EPD) pressure sensors were also tested. Figure 4g is the current–voltage (*I*–*V*) curves of the PI/FCNT(EPD) under various applied pressures. The *I*–*V* curves exhibit high linearity, revealing their excellent ohmic performance independent of the applied voltage. Figure 4h represents the current response of the PI/FCNT(EPD) pressure sensor over five on/off cycles under various external pressures (1.1, 1.3, 1.6, 2, and 7 MPa). The current experienced fast‐changing under the repeated pressure loading and relaxation cycles. Besides, the current remained unchanged during the pressure loading process, indicating the robust repeatability and excellent reliability of the PI/FCNT(EPD) pressure sensors independent of the applied pressures. We further explored the sensors’ durability by repeated loading and unloading pressure of 1.1 MPa for more than 4000 cycles, as shown in Figure 4i. It is found that the sensor maintained its function with minimal output signal degradation. This excellent endurance behavior can be attributed to the strong chemical interaction between PI and FCNT as well as the excellent compressibility of both PI fiber fabric and CNT. To demonstrate this, the compressive mechanical properties of the PI/FCNT(EPD) nanofibrous network under cyclic compressive stress–strain was characterized. Figure S24 (Supporting Information) shows cyclic compressive stress‐strain curves (1000 cycles) for the PI/FCNT(EPD) nanofibrous network at room temperature. Note that the tested PI/FCNT(EPD) nanofibrous network is encapsulated by PI film, and the encapsulation process is the same as that of the PI/FCNT(EPD) pressure sensor (see the Experimental section), which can better reflect the robustness of sensing performance of the PI/FCNT(EPD) pressure sensor. As shown in Figure S24 (Supporting Information), the PI/FCNT(EPD) nanofibrous network maintains over 91% of its initial maximum stress after 1000 cycles, demonstrating its superior compressive properties, ensuring a reliable and robust performance for long‐term applications. The proposed PI/FCNT (EPD) sensor device also exhibits excellent repeatability during cyclic bending test and winding test, as depicted in Figure S25 (Supporting Information). In addition, the fabricated sensor exhibits a fast rise time of 100 ms and a relaxation time of 80 ms (Figure 4j), which also indicates that the force unloading process is faster than that of the loading process. The response time is mainly determined by the elastic recovery of the PI fiber fabric. Hysteresis of the PI/FCNT(EPD) pressure sensor (linear region) is characterized as shown in Figure S26 (Supporting Information), and the sensor exhibits a hysteresis of 5.5%. The residual resistance is about 2.6%. The hysteresis and residual resistance may be due to the viscoelastic behavior of the PI. However, the hysteresis is relatively small, which may be attributed to the following two reasons: 1) large elastic modulus of the PI materials (PI fiber fabric and PI encapsulation layer); 2) the area of the electrode is independent of the sensing area (eliminating the effect of hysteretic behavior of the adhesive which is used to attach the sensing area and the electrode in the conventional vertical‐structured pressure sensor). Finally, the electric characteristics including *I*–*V* linearity and reliability of the sensor under 100 °C were also tested. As shown in Figure 4k,l, the results demonstrate high linearity and excellent reliability of the PI/FCNT(EPD) pressure sensor, further endowing it with high applicability in high‐temperature environments.

**Figure 5:**
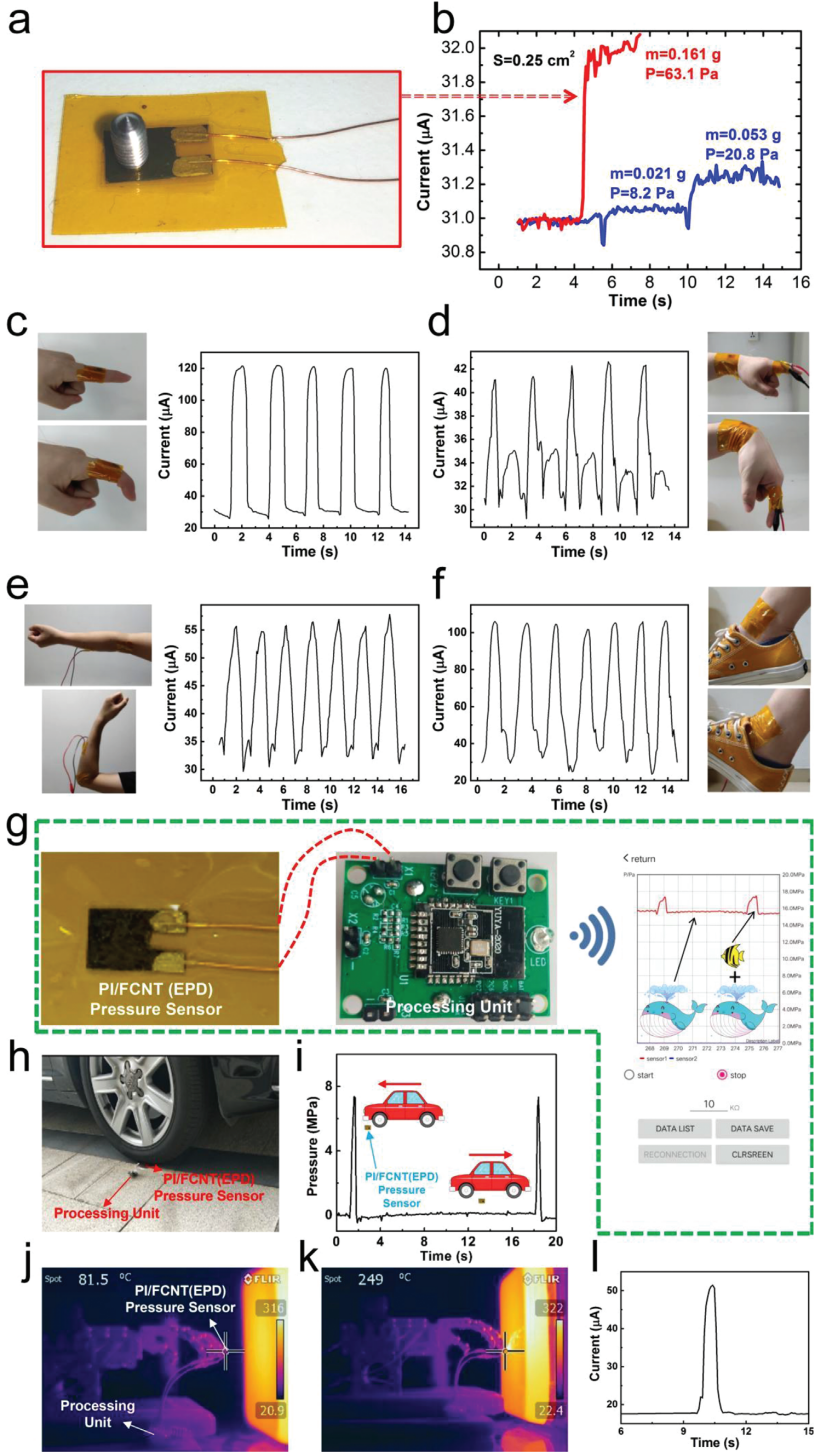
Application of the PI/FCNT(EPD) pressure sensor. a,b) Detection of faint pressure: optical image and current curve of the proposed PI/FCNT(EPD) pressure sensor pressed by a small meter screw (0.161 g, ≈63.1 Pa); Current curves of the proposed PI/FCNT (EPD) pressure sensor pressed by an aluminum particle with 0.021 g and 0.053 g (0.021 g+0.032 g), respectively. Faint pressures (≈8.2 Pa) can be detected by the PI/FCNT(EPD) pressure sensors. c–f) Detection of low pressure: monitoring finger bending, wrist movement, elbow bending, and ankle movement. g) A wireless, real‐time pressure monitoring system, including PI/FCNT(EPD) pressure sensor, processing unit, and mobile APP. Right of (g): detection of relatively low pressure under high pressure. h,i) Detection of high pressure: Real‐time pressure monitoring of the sensor device when subjected to high pressure applied by driving a car over the sensor device repeatedly. j–l) Intelligent robots “feel” the pressure under a high‐temperature environment: IR images of the mechanical hand with the PI/FCNT(EPD) pressure sensor as the mechanical hand get close to and touches the hot object (j, k). Signal response of the intelligent robot (with the integrated proposed sensor as the perception layer) during the “close, touch, feel and response” process.

These sensing characterizations and analyses demonstrate that the proposed PI/FCNT(EPD) pressure sensor exhibits superior sensing performance including wide linearity, exceptionally broad sensing range, and high‐temperature‐resistant properties. The wide linearity and relatively high sensitivity reveal that the resulting sensor can act as an ideal candidate to detect the faint pressure (<100 Pa), low‐pressure (in the range of KPa, such as human motions), and high pressure (in the range of MPa). To testify the sensing capability, we conducted the following experiments and further explored their applications in real life. First of all, as shown in **Figure** **5**a, a small meter screw (0.161 g, ≈63.1 Pa) is put on the sensor. The corresponding current change is displayed in Figure 5b (red line), which shows that such a tiny pressure change can be precisely detected. Moreover, the limit of detection of our sensor device was also measured to be ≈8.2 Pa, as depicted in Figure 5b (blue line, an aluminum particle, 0.021 g). Furthermore, a sharp current response for airflow again shows the capability of detecting the faint pressure (Movies S1 and S2, Supporting information). Second, the sensor's ability as a skin‐mountable human motion detector is explored. For the test, the fabricated PI/FCNT(EPD) pressure sensor was attached to various parts of the human body, as shown in Figure 5c–f and Figure S27 (Supporting Information). As a result, the fabricated PI/FCNT(EPD) pressure sensor is highly responsive to the repetitive dynamic flexion and straightening motions of the finger joint, wrist, elbow, and ankle. Besides, the cycling tests show that the response and relaxation behaviors are reproducible. The flexible PI/FCNT(EPD) pressure sensor can also be used to make a pulse sensor for the detection of the radial artery pulse (Figure S28, Supporting Information). The above results clearly suggest that the human motions can be identified with the PI/FCNT(EPD) pressure sensor, enabling its potential to monitor human physiological information in real time.

To further improve the practicability of the PI/FCNT(EPD) pressure sensors, a real‐time, wireless pressure monitoring system is developed, including data acquisition, wireless data transmission, and display via APP interface on a mobile phone, as displayed in Figure 5g. Here, the function of the processing unit shown in Figure 5g involves the data acquisition collected from the pressure sensor, analog‐digital conversion, and wireless data transmission. With the real‐time pressure monitoring system, two high‐pressure experiments were carried out: 1) the PI/FCNT(EPD) pressure sensors was first compressed to a reference high‐pressure of 15.8 MPa, followed by adding a metal with weighing about ≈3.8 kg, which is equivalent to a pressure increment of 1.5 MPa. The real‐time pressure monitoring during the metal loading and unloading process is displayed by the APP interface, as shown in the right session of Figure 5g. The results show that the PI/FCNT(EPD) pressure sensor can detect relatively small changes in pressure under extremely high pressures, even though the sensor is in a non‐linear state (as shown in Figure 4b). This significantly extends its available pressure sensing range, demonstrating its high potential for precise real‐time detection of extremely high pressure. 2) Another experiment is the pressure detection of a vehicle during the running process (Figure 5h). The PI/FCNT(EPD) pressure sensor is repeatedly rolled by the front wheel of a car. The recorded real‐time pressure data are collected from APP and displayed in Figure 5i. When the car is driven over the sensor, the pressure increases instantaneously and returns to its original state without any noticeable time lag. Besides, the sensor remained its function after the repeated rolling process. This greatly enables its application in the industry, such as safety monitoring of road, rail, bridge, and tunnel.

An ideal intelligent robot should not only require the basic capability of sensing force and pressure but also can collect some characteristic data in some harsh high‐temperature conditions (for example, firefighting). As a proof of concept, we designed the following experiments. We wrapped the PI/FCNT(EPD) pressure sensor in the fingertips of a mechanical hand while the mechanical hand was mounted on a computer‐controlled stepping motor. The mechanic hand with the conformally wrapped sensor moved forward to a high‐temperature object (>300 °C). When the sensor device touched the surface of the hot object, the output current increased. Here, a threshold current was set for the stepping motor. The stepping motor stopped and moved backward when the output current of the sensor device reached the threshold current. Figure 5j,k shows the IR images as the mechanic hand moved close to and touched the hot object, respectively. The temperature of the fingertip increased from the room temperature to 81.5 °C and 249 °C, respectively. Figure 5l shows the current responding curve of the PI/CNT(EPD) pressure sensor during the “close, touch, feel, and response” process. It is observed that the current rises steeply to the threshold value and recovers quickly, demonstrating its great potential to serve as a wearable and high sensitivity pressure sensor that is able to withstand harsh environments where high temperature is present.

**Figure 6:**
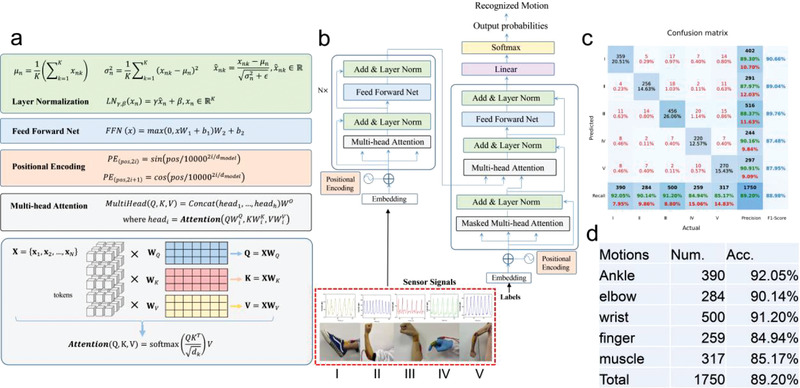
a,b) The recognition network architecture for our sensor‐based human action recognition (HAR). The main components include the layer normalization, feed forward network, positional encoding, and multihead attention. c) The confusion matrix for HAR classification. The labels (*I*–*V*) correspond to ankle/elbow/wrist/finger bending and muscle contractions, respectively. d) The statistics of the collected five human actions and the accuracy for each human action.

Deep learning has experienced rapid development in recent years for many missions, such as machine translation, natural language processing, as well as computer vision. Due to the sequence properties of the sensor signals, we use the network of the Transformer for sensor‐based human motion classification. The network architecture has been depicted in **Figure** **6**a,b, including its main components: layer normalization, feed forward network, positional encoding, and the multihead attention. By training the network model on our collected samples (1750 samples), we have achieved a recognition accuracy of 89.2% in cross‐validation of 5 different motions (ankle/elbow/wrist/finger bending, and muscle contractions) (Figure 6c,d), demonstrating its high applicability in real‐time practical human action recognition scenario through the help of the resulting PI/FCNT (EPD) pressure sensor.

**Figure 7:**
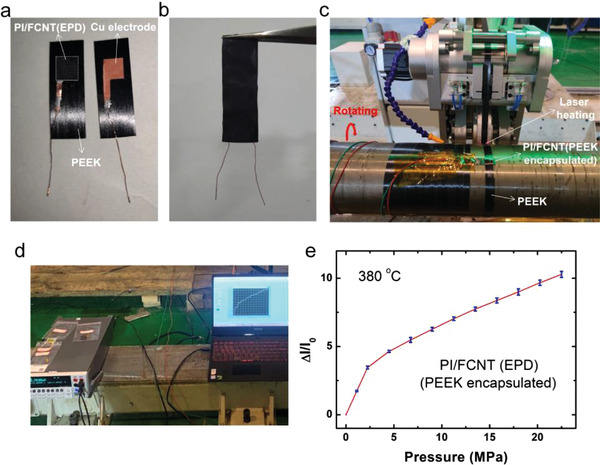
Application of the PI/FCNT(EPD) pressure sensor in both high temperature and high pressure environment. a,b) PI/FCNT (EPD) pressure sensor, the whole structure is PEEK‐Cu electrode‐PI/FCNT(EPD) film‐Cu electrode‐PEEK. c,d) Experimental setup for the real‐time detection of prestresses within the composite sheath of motor rotor under high temperature (380 °C). e) Relative current change versus the pressure applied to the PI/FCNT(EPD) pressure sensor during the winding process.

Poly (ether‐ether‐ketone) (PEEK) is a special engineering plastics and usually adopted as the protecting sheath of the motor rotor benefiting from its superior properties such as good resistant properties to chemical corrosion, et al. During the winding process (**Figure** **7**), the PEEKs will stick together under the high power laser heating process (380 °C), forming a huge tension imposed on the motor rotor. During the actual operation process, it is vital to detect the prestresses within the composite sheath of the motor rotor for the high‐speed permanent magnet motor rotor to ensure the safety of the whole system. For the prestresses monitoring sensor, several points should be addressed: 1) the sensor should be flexible, thin and can be integrated into the motor rotor; 2) the sensor should be high‐temperature resistant because there is a (laser) heating process; 3) the sensor can work at high‐pressure environment due to the large tension. Herein, we show that the fabricated PI/FCNT(EPD) pressure sensor can act as a promising candidate for the real‐time detection of the prestresses. Figure 7a,b shows the final PI/FCNT (EPD) pressure sensor with PEEK encapsulation. First of all, PI/FCNT(EPD) film, Cu and PEEK were adhered by conductive silver paste (left of Figure 7a, PEEK‐Cu‐PI/FCNT(EPD)). Right of Figure 7a shows the other side of the sensor (Cu‐PEEK), and the whole structure is PEEK‐Cu electrode‐PI/FCNT(EPD) film‐Cu electrode‐PEEK. PEEK‐Cu‐PI/FCNT(EPD) and Cu electrode‐PEEK were finally bonded together by welding the PEEK strips of both sides. Figure 7b shows the final encapsulated sensor device. Note that before the encapsulation process, the PI/FCNT (EPD) film was aged at 400 °C for 48 h. Figure 7c,d is the experimental setup for the PEEK winding process. In detail, the encapsulated sensor device was fixed on the curved motor rotor by high‐temperature glues. After the pressure sensor was fixed on the motor rotor (Figure 7c), the pressure applied on the sensor was defined as zero pressure. A PEEK strip was then fixed on the motor rotor, and then the motor rotor started to rotate. During the rotating process, the PEEK strip will impose the pressure on the sensor device (Figure 7c). To ensure the PEEK was firmly bonded on the motor rotor, a high tension and high temperature heating process was applied. The tension was applied to PEEK by the tension control system. The heating process was controlled by the high power laser (Laserline) and a closed‐loop temperature control system. Figure 6d depicts the real‐time current change of the sensor device during the PEEK rotating process. The pressure on the sensor can be obtained by the following equation: *P* = (tension/(PEEK_width_*PEEK_thickness_))*PEEK_thickness_/*R*_motor_, where the PEEK_width_ and PEEK_thickness_ are 12.7 and 0.145 mm, respectively, *R*_motor_ is the radius of the motor rotor (70 mm). At the beginning, the applied pressure was increased from 0 to 1000 N to 2000 N, and the applied pressure was ≈1.125 and ≈2.25 MPa, respectively. Afterwards, the applied pressure was ≈2.25 MPa for each circle. The *P*–*I* curve is shown in Figure 7e, and it is found that the linear range of the pressure sensor is partly reduced compared to Figure 4e due to the following several aspects: 1) initial pressure and normal/tensile stress are imposed on the sensor device during the welding and fixing process, respectively. Namely, part of the fibers come into contacts in this process, and 2) the high‐temperature aging process may result in loss of some bad contacting points. However, the cycling test results shown in Figure 7e indicate the robust repeatability of the proposed PI/FCNT (EPD) pressure sensor, demonstrating its potential use in both harsh high temperature and high‐pressure environment.

## 3 Discussion

The performances of the recently reported high‐performance pressure sensors are summarized in Table S1(Supporting Information). Superior comprehensive properties including thin, lightweight, broad working range, high‐temperature resistance, the capability of the detection of full‐range pressure (from < 100 Pa to ≈MPa) are found in the proposed PI/FCNT(EPD) pressure sensor. In addition, this large‐scale fabrication process of PI fiber fabrics, the proposed facile and robust EPD process, and the developed data transmission system are very beneficial to enable the technology transfer from the laboratory to the industry. The future scope for this study is to further increase the sensitivity. For example, appropriately reducing the Young's modulus of PI fiber fabrics (more point contacts variation, *∆*I/*I*_0_↑) via optimizing the solution formula and electrospinning process would address this issue, as well as ensuring broad linearity. Moreover, although the current application of the fabricated PI/FCNT(EPD) sensors focuses on the daily activities and harsh high temperature and high‐pressure condition, these thin, lightweight, flexible textile pressure sensors and the corresponding wireless pressure sensing system are ready to be used in practical biomedical applications, because they are resistant to most chemical attacks.

## 4 Conclusion

We have developed a simple, robust, and effective method via modification process and EPD method for fabricating high‐performance wearable pressure sensors. High‐density FCNT is evenly wrapped and chemically bonded to the PI fibers via the EPD process. Benefiting from the large yet appropriate compressible region of PI fiber fabric, abundant yet firm contacting points, point‐to‐point contacting mode within the hierarchical conductive network and high elastic modulus of both PI and CNT, the proposed PI/FCNT pressure sensor possesses the merits of thin, lightweight, wide linearity, exceptionally broad sensing range, and long‐term stability. Furthermore, thanks to the high‐temperature‐resistant properties of both PI and CNT, the PI/FCNT pressure sensor device still exhibits a sensitivity of 38.66 and 2.85 MPa^−1^ in the wide linear ranges of 0–36 KPa and 36 KPa–6.88 MPa. Besides, we have developed a real‐time, wireless pressure monitoring system and demonstrated that the pressure sensor could serve as an ideal platform to monitor the human physiological information, precisely detect extremely high pressure, and can be integrated into an intelligent mechanical hand to detect the contact force under the harsh high‐temperature environment.

## 5 Experimental Section

### Fabrication of PI/FCNT(EPD) Piezoresistive Pressure Sensor

FCNT was obtained by the modification process of CNT. CNT was purchased from XFNANO Co., Ltd. Next, 1.044 g CNTs were dispersed in 40 mL of the mixture of concentrated sulfuric acid (30 mL) and concentrated nitric acid (10 mL) in a flask. The mixture was then stirred using a magnetic stirrer at 150 °C for 1 h. The resulting dispersion was then diluted in water, and filtered repeatedly until the solution PH 7, followed by the drying process in vacuum at 40 °C overnight. The detailed fabrication process of PI fiber fabric and PI films can be found in our previous work.^[^ ^49^ ^]^ Briefly, poly(amic acid) (PAA) (10 wt%) was synthesized by an equimolar ratio of 3,3',4,4'‐biphenyltetracarboxylic dianhydride(BPDA) and 4,4'‐oxydianiline(ODA) in *N,N*'‐dimethylacetamide (DMAc) at room temperature for 24 h. The PAA solutions were diluted to 6 wt% by DMAc. The average electrical field was 100 kV m^‐1^ by imposing 20 kV electrical potential into a 20 cm gap between spinneret and collector. After removing the residual solvent in a vacuum oven at 75 °C for 8 h, the sample was imidized by the following processes: 1) heating up to 100 °C (5 °C min^‐1^, N_2_) and annealing for 1 h; 2) heating up to 200 °C (2 °C min^‐1^, N_2_) and annealing for 0.5 h; 3) heating up to 380 °C (2 °C min^‐1^, N_2_) and annealing for 0.3 h; 4) cooling down to the room temperature. Functional carbon nanotube dispersions were used to electrophoretically deposit nanotubes onto the PI fiber fabric. 0.1046 g FCNT was dispersed in 100 mL anhydrous alcohol and then stirred using magnetic for 7 h. The PI fiber fabric was placed in contact with a stainless steel cathode as the anode. The same stainless steel was placed a fixed distance from the anode. EPD process was carried out under direct current with the voltage of 30 V for 1 h. The PI/FCNT fiber fabric was then dried in an oven for 10 min at 60 °C. After the EPD and process, the PI/FCNT fiber fabric was cut into the designed concave shape, as shown in Figure 1a,c. Cu wire electrodes were adhered to the bulgy area of the sensor through the conductive silver paste.

### Fabrication of PI/CNT (stirring), PI/FCNT (stirring), and PI/CNT(EPD) Piezoresistive Pressure Sensor:PI/CNT (Stirring) Pressure Sensor

0.1046g CNT was dispersed in 100 mL anhydrous alcohol. Then the PI fiber fabric was immersed in the CNT dispersion and stirred using a magnetic stirrer for 7 h. The subsequent baking and tailoring process was the same as PI/FCNT (EPD). PI/FCNT (Stirring): Compared with the PI/CNT (Stirring) pressure sensor, besides the difference of the functional layer (FCNT instead of CNT), the other process was the same. PI/CNT (EPD): Compared with the PI/FCNT (EPD) pressure sensor, besides the difference of the functional layer (CNT instead of FCNT), the other process was the same.

### Encapsulation, Characterization, Test, and Simulation

First, the PI/FCNT (EPD) film was placed between two pieces of PI film, forming a sandwich structure. Then this sandwich structure was further encapsulated by the PI adhesive tape. The thickness of the encapsulated PI/FCNT (EPD) pressure sensor was ≈0.33 mm (Figure S29, Supporting Information). The 2D and 3D morphologies of the PI fiber fabric and PI/FCNT(EPD) were characterized using SEM (AEISS SUPRA55) and KEYENCE VK‐X1000. The morphologies of CNT and FCNT were characterized using TEM (JEM‐3200FS) with 200 kV. CNT(FCNT) powder was dispersed in the ethanol solution and a dilute CNT(FCNT) solution was dripped on the TEM grid, followed by vacuum drying at 30 °C. For Raman test, Raman analysis was carried out on a Witec CRM200 backscattering Raman system. Raman system was equipped with a solid‐state YAG laser at 2.33 eV (532 nm). An objective (Olympus, 100 × /NA0.95)) was used for focusing the laser beam (laser power ≈ 1.5 mW). CNT and FCNT powder, PI and PI/FCNT film were prepared for the Raman characterization. The compression test of the PI fiber fabrics and PI/FCNT(EPD) was carried out on a universal testing machine (SHIMADZU AG‐X Plus 1 kN). For XPS measurement, ESCALAB 250Xi was used. The sample size of PI and PI/FCNT (EPD) film were both 5 mm × 5 mm. The PI (PI/FCNT(EPD)) was adhered to the stage using conductive adhesive. The vacuum is below 10–9 Pa, monochromatic Al Ka X‐ray source with the voltage of 15 kV was applied for the spectral acquisition. For FTIR measurement, Invenio R was used. For CNT and FCNT analysis, CNT(FCNT) powder was put in the build‐in small grooves. The probe is used to press the powders, and the distance between the probe and the powder is less than 1 mm. For PI and PI/FCNT(EPD) analysis, the PI and PI/FCNT(EPD) film with the size of 5 mm × 5 mm were put on the build‐in stages. The distance between the probe and powder is less than 1 mm. TGA analysis was conducted on a thermogravimetric analyzer (Mettler Toledo, Switzerland) from room temperature to 800 °C. A semiconductor parameter analyzer Keithley 2400 was employed to measure the current–time (*I*–*t*) characteristics of the pressure sensors. The cycling performance of the pressure sensor was determined using a pressure meter (Dongguan Zhiqu Precision Instrument Co., Ltd). Oscilloscope (LUCK‐3, digital storage oscilloscope, Chengdu Rongte Instrument Co., Ltd.) was used to measure the response and recovery time. In order to theoretically study the sensing performance of the pressure sensors, Creo and Ansys software were employed in the simulation. PI/CNT models were designed and built via Creo software, then the established models were imported into Ansys software for further analysis. The PI fiber was modeled as a homogeneous isotropic elastic material with Young's modulus *E* = 2.5 GPa, and Poisson's ratio *v* = 0.34. For high‐temperature *P*–*I* and other electric characterization, the PI/FCNT (EPD) film was baked at 200 °C for 48 h for the aging process.

### Statistical Analysis

All shown data are representative for the samples. The sample size is provided in the subsection entitled “Encapsulation, characterization, test, and simulation” at the Experimental Section. All plotting and analysis were conducted with Origin 8 software. Data in Figure 7e are presented as the mean ± S.D. Ansys and Creo was utilized for simulation.

### Testing the Devices on Human Bodies

All human subjects involved in the tests provided informed consent, and the study was approved by the Shenzhen Institute of Advanced Technology, Chinese Academy of Sciences. The skin shown in the figures and video are those of G.X. and Y. Z., who have given his consent to publish these images and movies.

## Conflict of Interest

The authors declare no conflict of interest.

## Supporting information

Supporting Information

Supplemental Movie 1

Supplemental Movie 2

## Data Availability

The data that support the findings of this study are available from the corresponding author upon reasonable request.

## References

[1] J. Kim , E.n‐F. Chou , J. Le , S. Wong , M. Chu , M. Khine , Adv. Healthcare Mater. 2019, 8, 1900109. 10.1002/adhm.201900109 31033256

[2] G. Schwartz , B. C.‐K. Tee , J. Mei , A. L. Appleton , D.o H. Kim , H. Wang , Z. Bao , Nat. Commun. 2013, 4, 1859. 23673644 10.1038/ncomms2832

[3] C. M. Boutry , A. Nguyen , Q. O. Lawal , A. Chortos , S. Rondeau‐Gagné , Z. Bao , Adv. Mater. 2015, 27, 6954. 26418964 10.1002/adma.201502535

[4] X. Tang , C. Wu , L. Gan , T. Zhang , T. Zhou , J. Huang , H. Wang , C. Xie , D. Zeng , Small 2019, 15, 1804559. 10.1002/smll.201804559 30714294

[5] D. Rus , M. T. Tolley , Nature 2015, 521, 467. 26017446 10.1038/nature14543

[6] M. Liu , X. Pu , C. Jiang , T. Liu , X. Huang , L. Chen , C. Du , J. Sun , W. Hu , Z. L. Wang , Adv. Mater. 2017, 29, 1703700. 10.1002/adma.201703700 28949422

[7] X. Wang , L. Dong , H. Zhang , R. Yu , C. Pan , Z. L. Wang , Adv. Sci. 2015, 2, 1500169. 10.1002/advs.201500169 PMC5115318 27980911

[8] X. Wang , Y. Gu , Z. Xiong , Z. Cui , T. Zhang , Adv. Mater. 2014, 26, 1336. 24347340 10.1002/adma.201304248

[9] B. Zhu , Y. Ling , L. W. Yap , M. Yang , F. Lin , S. Gong , Y. Wang , T. An , Y. Zhao , W. Cheng , ACS Appl. Mater. Interfaces 2019, 11, 29014. 31322334 10.1021/acsami.9b06260

[10] K. Meng , S. Zhao , Y. Zhou , Y. Wu , S. Zhang , Q. He , X. Wang , Z. Zhou , W. Fan , X. Tan , J. Yang , J. Chen , Matter 2020, 2, 896.

[11] X. Peng , K. Dong , C. Ye , Y. Jiang , S. Zhai , R. Cheng , D.i Liu , X. Gao , J. Wang , Z. L. Wang , Sci. Adv. 2020, 6, eaba9624. 32637619 10.1126/sciadv.aba9624 PMC7319766

[12] L. Lin , Y. Xie , S. Wang , W. Wu , S. Niu , X. Wen , Z. L. Wang , ACS Nano 2013, 7, 8266 23957827 10.1021/nn4037514

[13] J. Chen , K. Han , J. Luo , L. Xu , W. Tang , Z. L. Wang , Nano Energy 2020, 77, 105171.

[14] N. Bai , L. Wang , Q.i Wang , J. Deng , Y. Wang , P. Lu , J. Huang , G. Li , Y. Zhang , J. Yang , K. Xie , X. Zhao , C. F. Guo , Nat. Commun. 2020, 11, 209. 31924813 10.1038/s41467-019-14054-9 PMC6954251

[15] Y. Luo , J. Shao , S. Chen , X. Chen , H. Tian , X. Li , L. Wang , D. Wang , B. Lu , ACS Appl. Mater. Interfaces 2019, 11, 17796. 31007008 10.1021/acsami.9b03718

[16] Y. Huang , X. Fan , S. ‐. C. Chen , N.i Zhao , Adv. Funct. Mater. 2019, 29, 1808509.

[17] Y.e Yang , H. Pan , G. Xie , Y. Jiang , C. Chen , Y. Su , Y. Wang , H. Tai , Sens. Actuators, A 2020, 301, 111789.

[18] Y. Ma , N. Liu , L. Li , X. Hu , Z. Zou , J. Wang , S. Luo , Y. Gao , Nat. Commun. 2017, 8, 1207. 29089488 10.1038/s41467-017-01136-9 PMC5663936

[19] C. Luo , N. Liu , H. Zhang , W. Liu , Y. Yue , S. Wang , J. Rao , C. Yang , J. Su , X. Jiang , Y. Gao , Nano Energy 2017, 41, 527.

[20] L. Gao , C. Zhu , L. Li , C. Zhang , J. Liu , H. ‐. D. Yu , W. Huang , ACS Appl. Mater. Interfaces 2019, 11, 25034. 31268663 10.1021/acsami.9b07465

[21] S. K. Ghosh , S. Chakraborty , A. Jamthe , D. P. Agrawal , in IEEE Tenth Int. Conf. on Wireless and Optical Communications Networks (WOCN), IEEE, Piscataway, NJ 2013, pp. 1–6.

[22] A. Coca , R. J. Roberge , W. J. Williams , D. P. Landsittel , J. B. Powell , A. Palmiero , J. Occup. Environ. Hyg. 2009, 7, 109. 10.1080/15459620903455722 20017053

[23] M. Fu , J. Zhang , Y. Jin , Y. Zhao , S. Huang , C. F. Guo , Adv. Sci. 2020, 7, 2000258. 10.1002/advs.202000258 PMC7507114 32995117

[24] Y. Hu , L. Li , W. Guo , S. Wang , Symmetry 2021, 13, 2161.

[25] M. Hooker , C. Hazelton , K. Kano , High‐Temperature Motor Windings for Downhole Pumps Used in Geothermal Energy Production. No. DOE/GO/18183‐1. Composite Technology Development, Inc., Lafayette, CO, USA, 2010, https://www.energy.gov/eere/geothermal/downloads/high-temperature-motor-windings-downhole-pumps-used-geothermal-energy. (accessed: January 2022).

[26] R. Jiang , L. Mei , Q. M. Zhang , MRS Adv. 2016, 1, 1525.

[27] B. Zhu , Z. Niu , H. Wang , W. R.u Leow , H. Wang , Y. Li , L. Zheng , J. Wei , F. Huo , X. Chen , Small 2014, 10, 3625. 24895228 10.1002/smll.201401207

[28] S. Peng , P. Blanloeuil , S. Wu , C. H. Wang , Adv. Mater. Interfaces 2018, 5, 1800403.

[29] Z. Wang , S. Wang , J. Zeng , X. Ren , A. J. Y. Chee , B. Y. S. Yiu , W. C. Chung , Y. Yang , A. C. H. Yu , R. C. Roberts , A. C. O. Tsang , K. W. Chow , P. K. L. Chan , Small 2016, 12, 3827. 27280488 10.1002/smll.201601419

[30] M. Jian , K. Xia , Q.i Wang , Z. Yin , H. Wang , C. Wang , H. Xie , M. Zhang , Y. Zhang , Adv. Funct. Mater. 2017, 27, 1606066.

[31] K. He , Y. Hou , C. Yi , N. Li , F. Sui , B. Yang , G. Gu , W. Li , Z. Wang , Y. Li , G. Tao , L. Wei , C. Yang , M. Chen , Nano Energy 2020, 73, 104743.

[32] M. Chen , K. Li , G. Cheng , K. He , W. Li , D. Zhang , W. Li , Y. Feng , L. Wei , W. Li , G. Zhong , C. Yang , ACS Appl. Mater. Interfaces 2019, 11, 2551. 30576104 10.1021/acsami.8b20284

[33] C. Yi , Y. Hou , K. He , W. Li , N. Li , Z. Wang , B. Yang , S. Xu , H. Wang , C. Gao , Z. Wang , G. Gu , Z. Wang , L. Wei , C. Yang , M. Chen , ACS Appl. Mater. Interfaces 2020, 12, 19563. 32301610 10.1021/acsami.0c02774

[34] W. Li , K. He , D. Zhang , N. Li , Y. Hou , G. Cheng , W. Li , F. Sui , Y. Dai , H. Luo , Y. Feng , L. Wei , W. Li , G. Zhong , M. Chen , C. Yang , ACS Appl. Energy Mater. 2019, 2, 2803.

[35] D.‐J. Liaw , K.‐L. Wang , Y.‐C. Huang , K.‐R. Lee , J. ‐Y. Lai , C.‐S. Ha , Prog. Polym. 2012, 37, 907.

[36] I. Gouzman , E. Grossman , R. Verker , N. Atar , A. Bolker , N. Eliaz , Adv. Mater. 2019, 31, 1807738. 10.1002/adma.201807738 30803081

[37] X. Chen , H.u Liu , Y. Zheng , Y. Zhai , X. Liu , C. Liu , L. Mi , Z. Guo , C. Shen , ACS Appl. Mater. Interfaces 2020, 11, 42594. 10.1021/acsami.9b14688 31618002

[38] Y. Jeong , J. Gu , J. Byun , J. Ahn , J. Byun , K. Kim , J. Park , J. Ko , J.‐H.o Jeong , M. Amjadi , I. Park , Adv. Healthcare Mater. 2021, 10, 2001461. 10.1002/adhm.202001461 33694309

[39] L. Vaisman , H. D. Wagner , G. Marom , Adv. Colloid Interface Sci. 2006, 128, 37. 17222381 10.1016/j.cis.2006.11.007

[40] J. Hilding , E. A. Grulke , Z. George Zhang , F. Lockwood , J. Dispersion Sci. Technol. 2007, 24, 1.

[41] O. Rodríguez‐Uicab , C. Martin‐Barrera , A. May‐Pat , A. Can‐Ortiz , P.i Gonzalez‐Chi , F. Avilés , J. Intell. Mater. Syst. Struct. 2019, 30, 1527.

[42] Q.i An , A. N. Rider , E. T. Thostenson , ACS Appl. Mater. Interfaces 2013, 5, 2022. 23379418 10.1021/am3028734

[43] G. J. Ehlert , H. A. Sodano , J. Intell. Mater. Syst. Struct. 2014, 25, 2117.

[44] I. D. Rosca , F. Watari , M. Uo , T. Akasaka , Carbon 2005, 43, 3124.

[45] F. Avilés , A. Ponce , J. V. Cauich‐Rodríguez , G. T. Martínez , Fullerenes, Nanotubes, Carbon Nanostruct. 2012, 20, 49.

[46] S. Osswald , M. Havel , Y. Gogotsi , J. Raman Spectrosc. 2007, 38, 728.

[47] Y. Qin , Q. Peng , Y. Ding , Z. Lin , C. Wang , Y. Li , F. Xu , J. Li , Y.e Yuan , X. He , Y. Li , ACS Nano 2015, 9, 8933. 26301319 10.1021/acsnano.5b02781

[48] S. Ramakrishnan , M. Dhakshnamoorthy , E. J. Jelmy , R. Vasanthakumari , N. K. Kothurkar , RSC Adv. 2014, 19, 9743. 10.1166/jnn.2014.8713 24757974

[49] P. Ma , W. Li , C. Yi , C. Dai , H. Luo , C. Yang , AIP Adv. 2019, 9, 045024.

