# Supplementary material for: Hierarchical Network Enabled Flexible Textile Pressure Sensor with Ultrabroad Response Range and High‐Temperature Resistance
Source: Adv Sci (Weinh). 2022 Mar 14;9(14):2105738. doi: 10.1002/advs.202105738 (PMC9108605; doi:10.1002/advs.202105738)
Supplement: Supplementary file 1 — Supporting Information [file ADVS-9-2105738-s001.pdf]

ADVANCED SCIENCE
Open Access

Supporting Information

for *Adv. Sci.*, DOI 10.1002/advs.202105738

Hierarchical Network Enabled Flexible Textile Pressure Sensor with Ultrabroad Response Range and High-Temperature Resistance

Meiling Jia, Chenghan Yi, Yankun Han, Lei Wang, Xin Li, Guoliang Xu, Ke He, Nianci Li, Yuxin Hou, Zhongguo Wang, Yuanhao Zhu, Yuanao Zhang, Mingzhu Hu, Ran Sun, Peifei Tong, Jiawei Yang, Yougen Hu, Zhixun Wang, Weimin Li, Wenjie Li, Lei Wei, Chunlei Yang* and Ming Chen**

WILEY-VCH

ADVANCED SCIENCE
Open Access

Supporting Information

for *Adv. Sci.*, DOI: 10.1002/advs.202105738

Hierarchical network enabled flexible textile pressure sensor with ultra-broad response range and high-temperature resistance

Meiling Jia, Chenghan Yi, Yankun Han, Lei Wang, Xin Li, Guoliang Xu, Ke He, Nianci Li, Yuxin Hou, Zhongguo Wang, Yuanhao Zhu, Yuanao Zhang, Mingzhu Hu, Ran Sun, Peifei Tong, Jiawei Yang, Yougen Hu, Zhixun Wang, Weimin Li, Wenjie Li, Lei Wei, Chunlei Yang* and Ming Chen**

Supporting Information

Hierarchical network enabled flexible textile pressure sensor with ultra-broad response range and high-temperature resistance

Meiling Jia[1,2#], Chenghan Yi[1,2#], Yankun Han[1,2#], Lei Wang[1#], Xin Li[1,2], Guoliang Xu[1,2], Ke He[1,2], Nianci Li[1,3], Yuxin Hou[1,3], Zhongguo Wang[1,2], Yuanhao Zhu[1,2], Yuanao Zhang[1,2], Ran Sun[1], Peifei Tong[1,2], Jiawei Yang[1,2], Yougen Hu[4], Zhixun Wang[5], Weimin Li[1], Wenjie Li[1], Lei Wei[5], Chunlei Yang[1,3*] and Ming Chen[1,3*]*

[1]Center for Photonics Information and Energy Materials, Shenzhen Institute of Advanced Technology, Chinese Academy of Sciences, Shenzhen 518055, People's Republic of China

[2]Department of Nano Science and Technology Institute, University of Science and Technology of China, Suzhou 215123, People's Republic of China

[3]School of Computer and Control Engineering, University of Chinese Academy of Sciences, Beijing 100049, People's Republic of China

[4]Shenzhen Institutes of Advanced Electronic Materials, Shenzhen Institutes of Advanced Technology, Chinese Academy of Sciences, Shenzhen518055, People's Republic of China

[5]School of Electrical and Electronic Engineering, Nanyang Technological University, 50 Nanyang Avenue, 639798, Singapore

[#]These authors contributed equally to this work.

*Corresponding authors; email: wei.lei@ntu.edu.sg; cl.yang@siat.ac.cn; ming.chen2@siat.ac.cn.

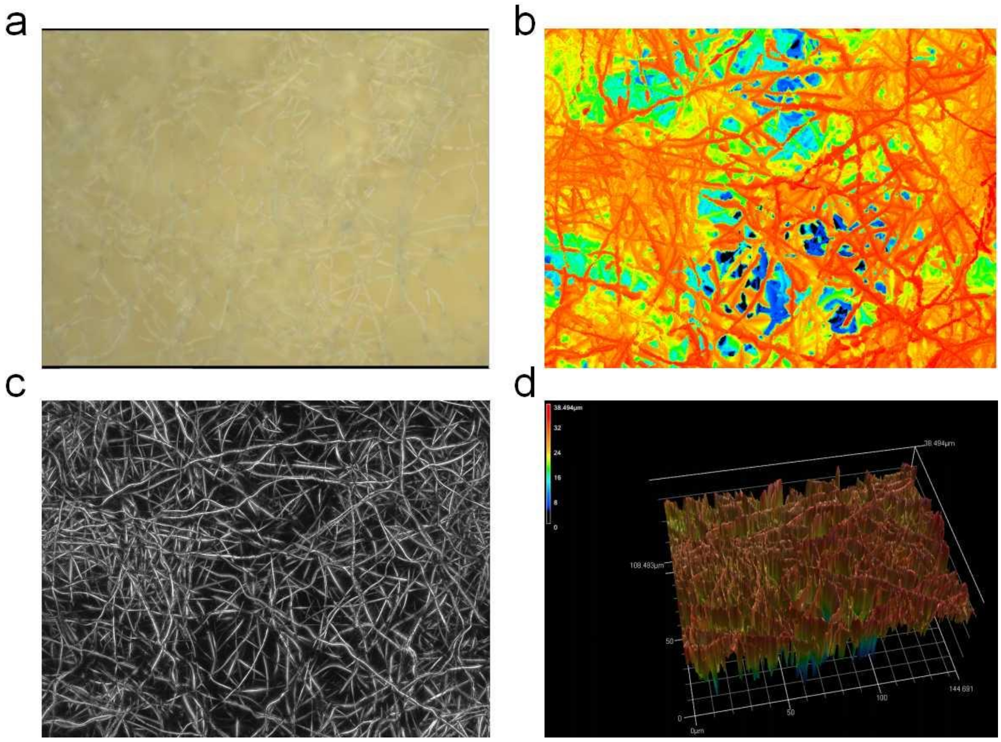

Figure S1. (a) The optical image of PI fiber fabric. (b,c) The 2D view of PI fiber fabric. (d) The 3D stereoscopic topography of the PI fiber fabric.

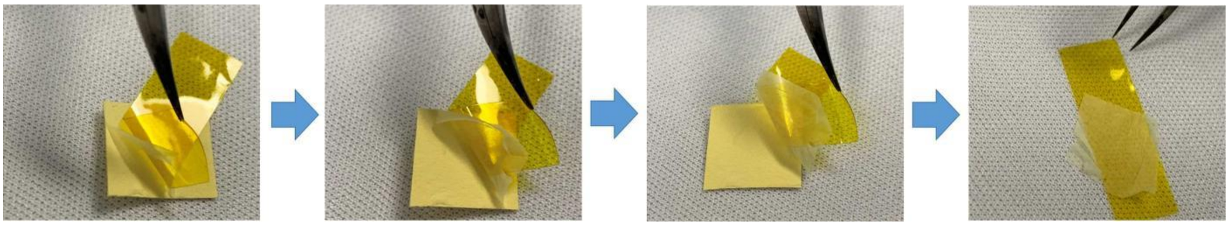

Figure S2. PI film is consisted of multi-layered PI fibers.

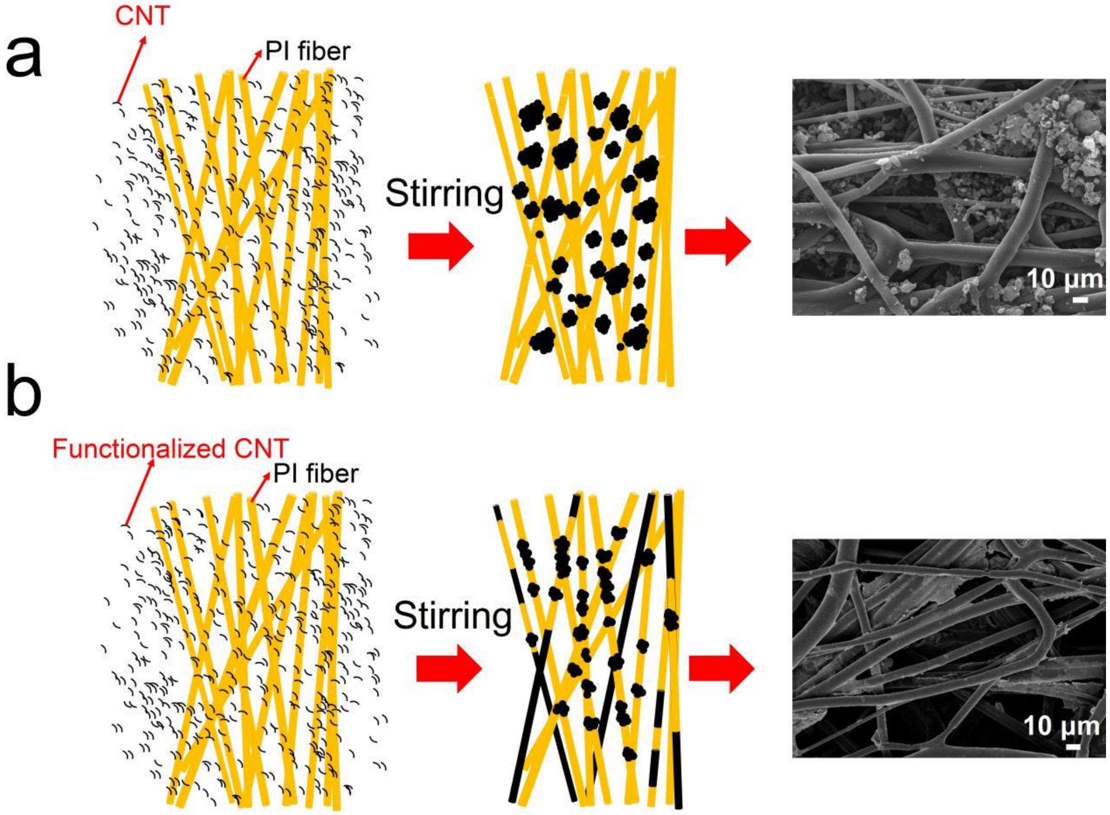

Figure S3. PI/CNT(Stirring) and PI/FCNT(Stirring) pressure sensors. (a)Schematic illustration of the doping mechanism for PI/CNT (Stirring) pressure sensors and the corresponding top-view SEM images of the PI/CNT(Stirring). (b) Schematic illustration of the doping mechanism for PI/FCNT (Stirring) pressure sensors and the corresponding top-view SEM images of the PI/FCNT(Stirring).

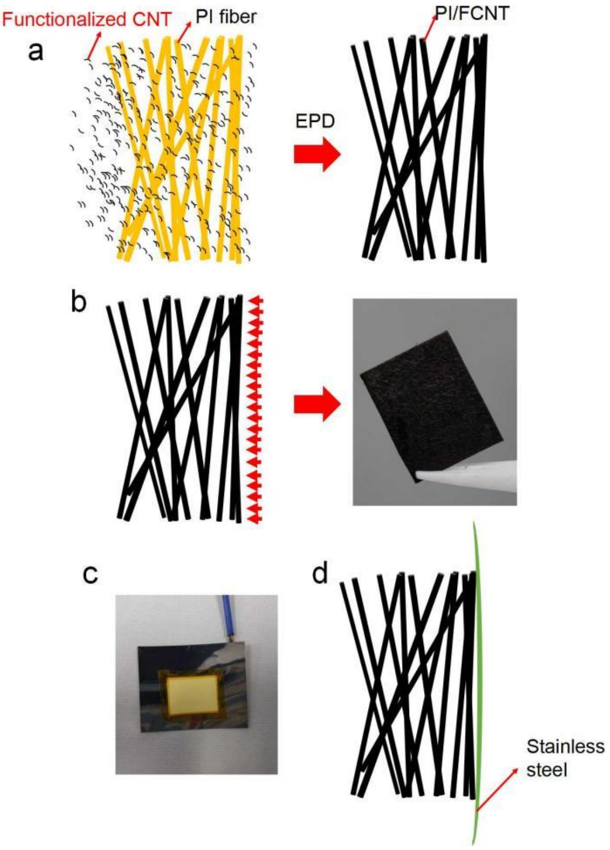

Figure S4. PI/FCNT(EPD) pressure sensor. (a)Schematic illustration of the doping mechanism for PI/FCNT (EPD) pressure sensor. (b) Optical image of the surface of the PI/FCNT(EPD). The FCNT is uniformly coated onto the fiber surface, even for the fibers adhered to the anode. (c-d) Image of the PI fiber fabric glued on the stainless steel anode, and the tape is rounded the edges of PI fiber fabric. After immersing the PI fiber fabric into the solution, most of the PI fiber fabric areas are exposed to the FCNT solution. During the EPD process, the FCNT can still be uniformly deposited onto the back surface of the PI fiber fabric.

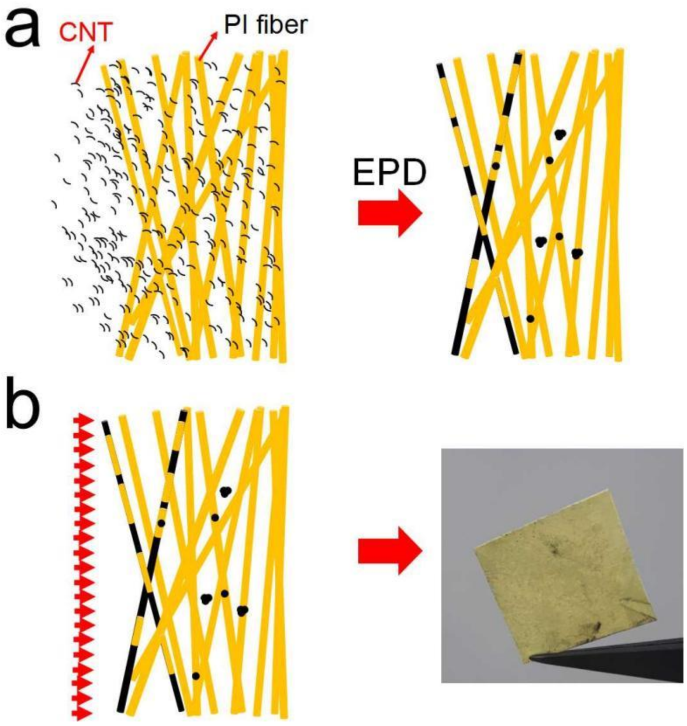

Figure S5. PI/CNT(EPD) pressure sensor. (a)Schematic illustration of the doping mechanism for PI/CNT (EPD) pressure sensor. (b) Optical image of the surface of the PI/CNT(EPD). We only observed a thin layer of CNT adhered to one surface (away from the anode) of the PI fiber fabric.

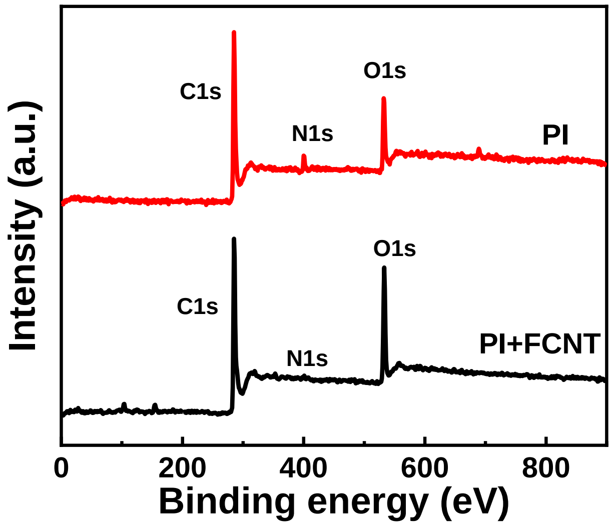

Figure S6. XPS survey spectra of PI and PI/FCNT(EPD).

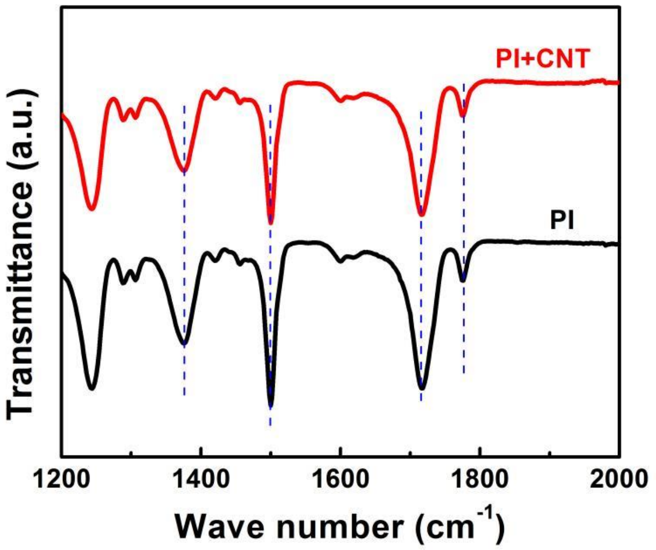

Figure S7. FT-IR spectra of PI and PI/CNT (Stirring).

FEM Simulation

In order to better explain the pressure mechanism of the sensor and theoretically explain the working principle of the pressure sensor, we adopted finite element simulation to simulate the experimental results. We built the geometric models using the Workbench Geometry model, as shown in Figure S8 (PI/FCNT(EPD)). Fourteen fibers with diameter of five microns are used to form a three-layer structure. At the same time, two plates were established to facilitate the application of boundary conditions. We used the “Static Structure” analysis system to carry out mechanical simulation for this geometric structure. The details of Static Structural is presented in Figure S9, showing the added fixed support in the bottom plate and a displacement condition of the top plate to apply static force to the intermediate fibers. Figure S10 shows the details of the contact situation we set. We used the time-contact area to describe some functions, because the displacement of the top plate is moving constantly with time. The function is displacement=2 µm × time (0<time<1s). The inputs are the displacement and applied force, and the outputs are the contacting area between the CNTs. The element was a hexagon grid. We adopted the Normal Lagrange Formulation to solve the contacting issues (Figure S10). In the Normal Lagrange Method, the contacting pressure was viewed as one degree of freedom, and no penetrating between the fibers was assumed, the calculated results had higher precision. The description for the contacting area is the total area of the elements that are in contact, we use "on Gauss point method" to detect the connection and ensure more accurate calculation results. The contacting area is defined by the formulation as: "detected total finite element Gauss points * (element size/element Gauss points). For PI/FCNT(EPD), the contacting area change was calculated between the PI/FCNT fibers to PI/FCNT fibers. For PI/CNT(stirring), the contacting area change was calculated between the CNT cluster to CNT cluster. For PI/FCNT(stirring), the contacting area change was calculated between the CNT cluster to PI/FCNT fibers. In the simulation, the CNT cluster was modeled as a semisphere attached to the PI fibers (Figure S11), and the quantity of CNT clusters (semisphere) for PI/CNT (stirring) was more than that of PI/FCNT(stirring), according to the SEM observations (Figure 1).

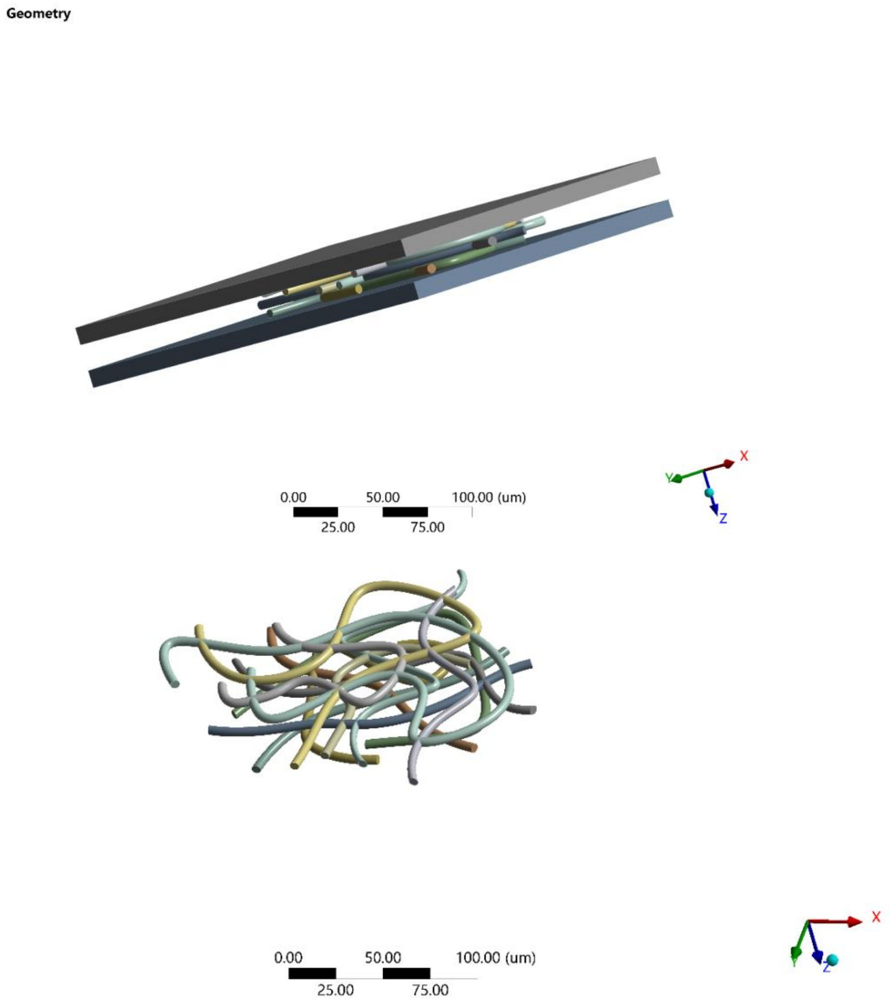

Figure S8. Geometry model of fibers for simulation.

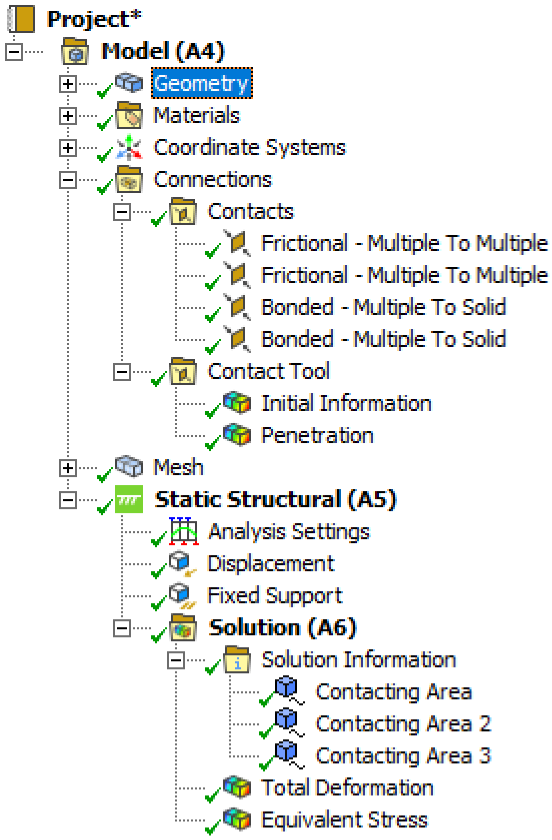
Project*
Model (A4)
Geometry
Materials
Coordinate Systems
Connections
Contacts
Frictional - Multiple To Multiple
Frictional - Multiple To Multiple
Bonded - Multiple To Solid
Bonded - Multiple To Solid
Contact Tool
Initial Information
Penetration
Mesh
Static Structural (A5)
Analysis Settings
Displacement
Fixed Support
Solution (A6)
Solution Information
Contacting Area
Contacting Area 2
Contacting Area 3
Total Deformation
Equivalent Stress

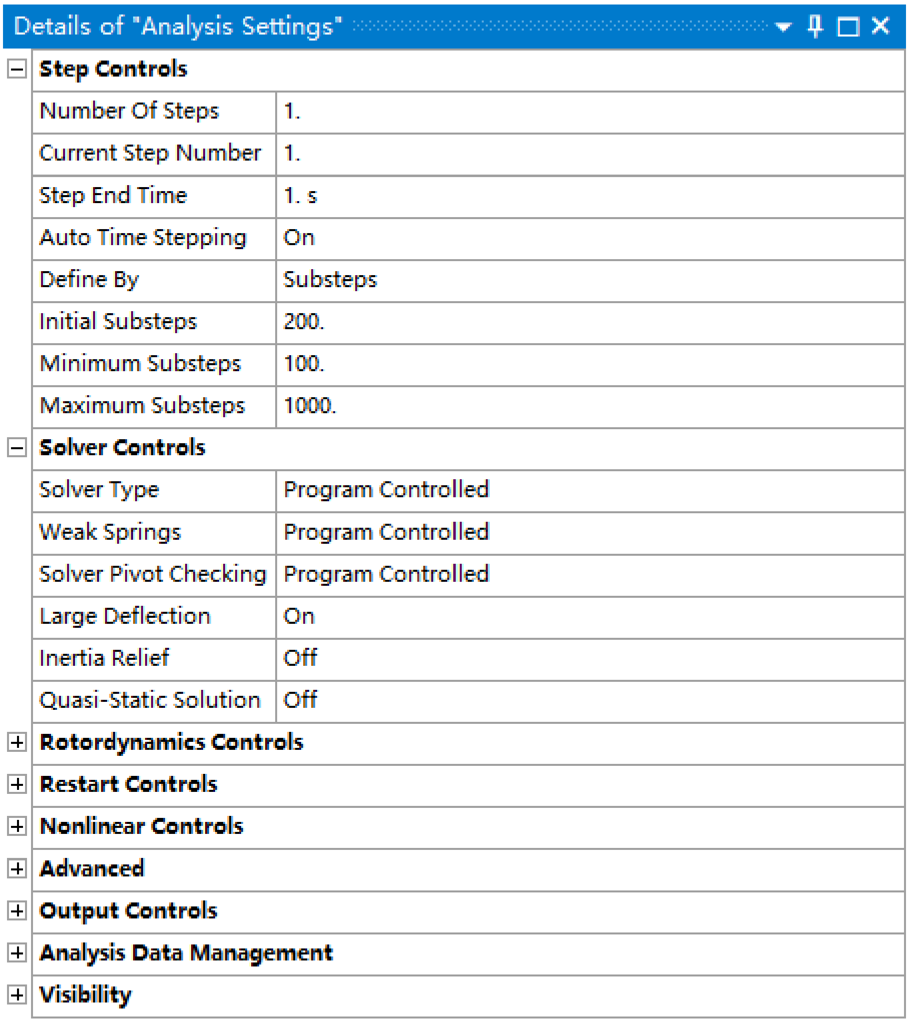

Details of "Analysis Settings"

**Step Controls**	
Number Of Steps	1.
Current Step Number	1.
Step End Time	1. s
Auto Time Stepping	On
Define By	Substeps
Initial Substeps	200.
Minimum Substeps	100.
Maximum Substeps	1000.
**Solver Controls**	
Solver Type	Program Controlled
Weak Springs	Program Controlled
Solver Pivot Checking	Program Controlled
Large Deflection	On
Inertia Relief	Off
Quasi-Static Solution	Off
**Rotordynamics Controls**	
**Restart Controls**	
**Nonlinear Controls**	
**Advanced**	
**Output Controls**	
**Analysis Data Management**	
**Visibility**	

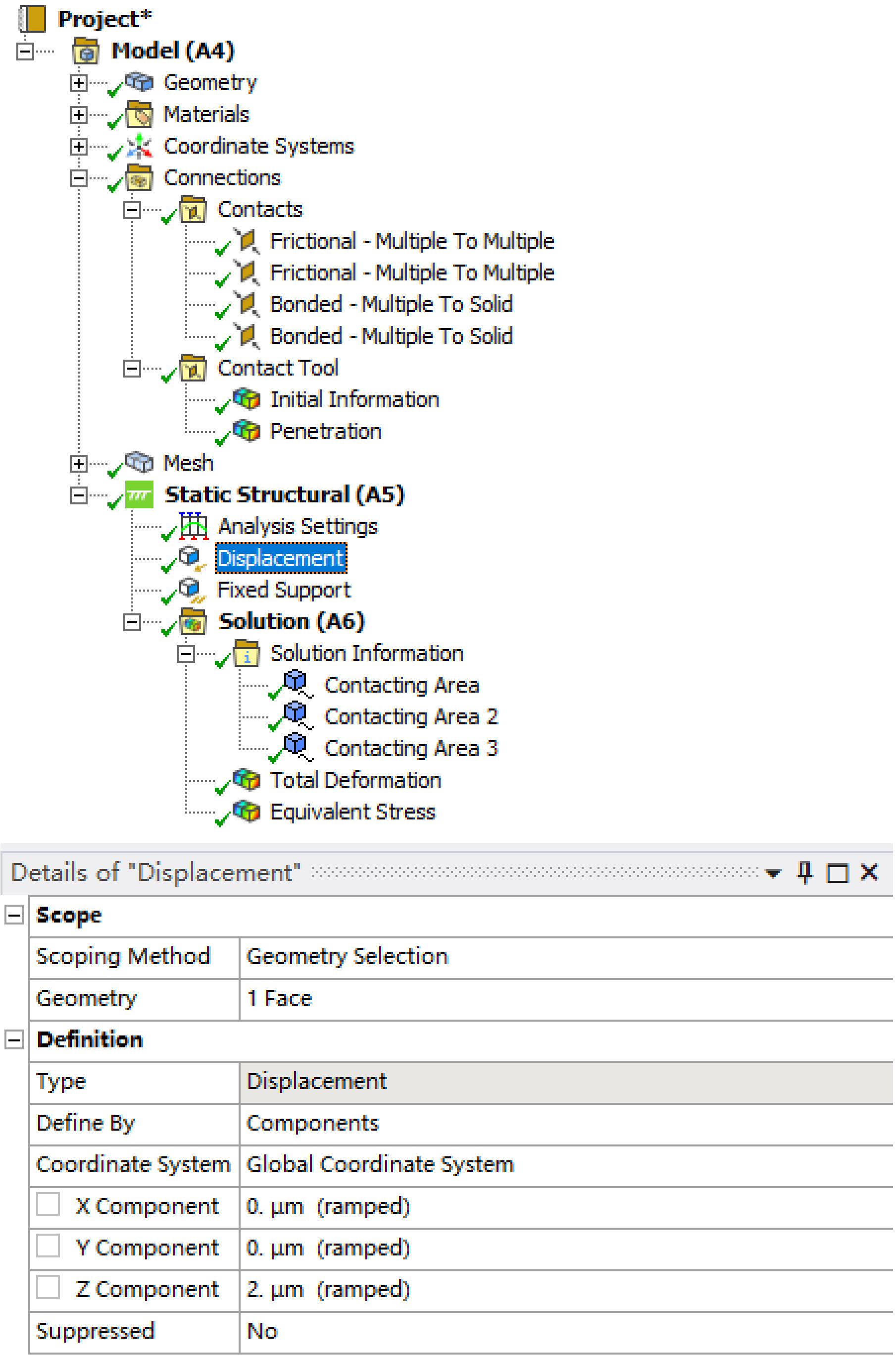
Project*
Model (A4)
Geometry
Materials
Coordinate Systems
Connections
Contacts
Frictional - Multiple To Multiple
Frictional - Multiple To Multiple
Bonded - Multiple To Solid
Bonded - Multiple To Solid
Contact Tool
Initial Information
Penetration
Mesh
Static Structural (A5)
Analysis Settings
Displacement
Fixed Support
Solution (A6)
Solution Information
Contacting Area
Contacting Area 2
Contacting Area 3
Total Deformation
Equivalent Stress
Details of "Displacement"
Scope
Scoping Method
Geometry Selection
Geometry
1 Face
Definition
Type
Displacement
Define By
Components
Coordinate System
Global Coordinate System
X Component
0. µm (ramped)
Y Component
0. µm (ramped)
Z Component
2. µm (ramped)
Suppressed
No

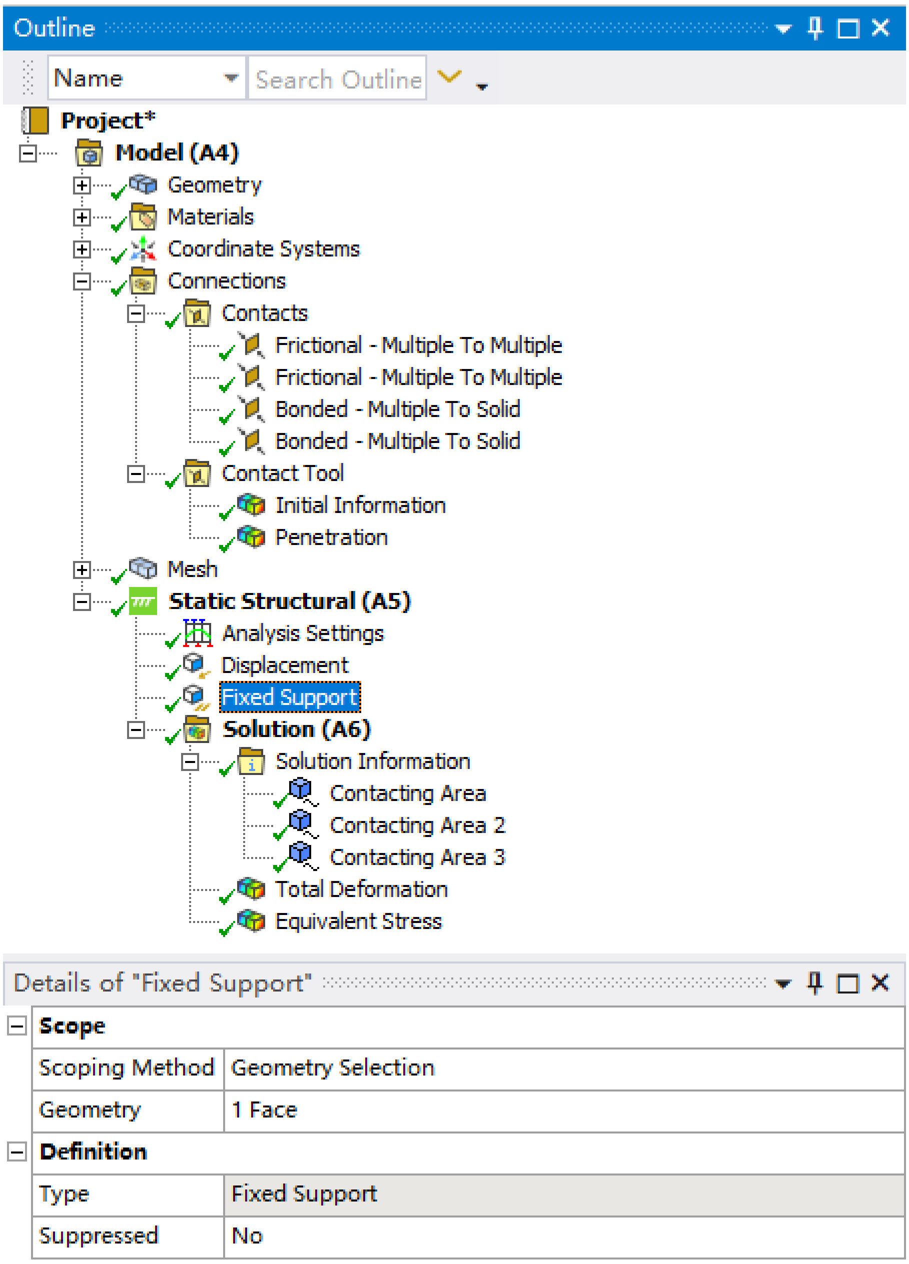

Figure S9. Parameter display regarding the boundary conditions in ANSYS software.

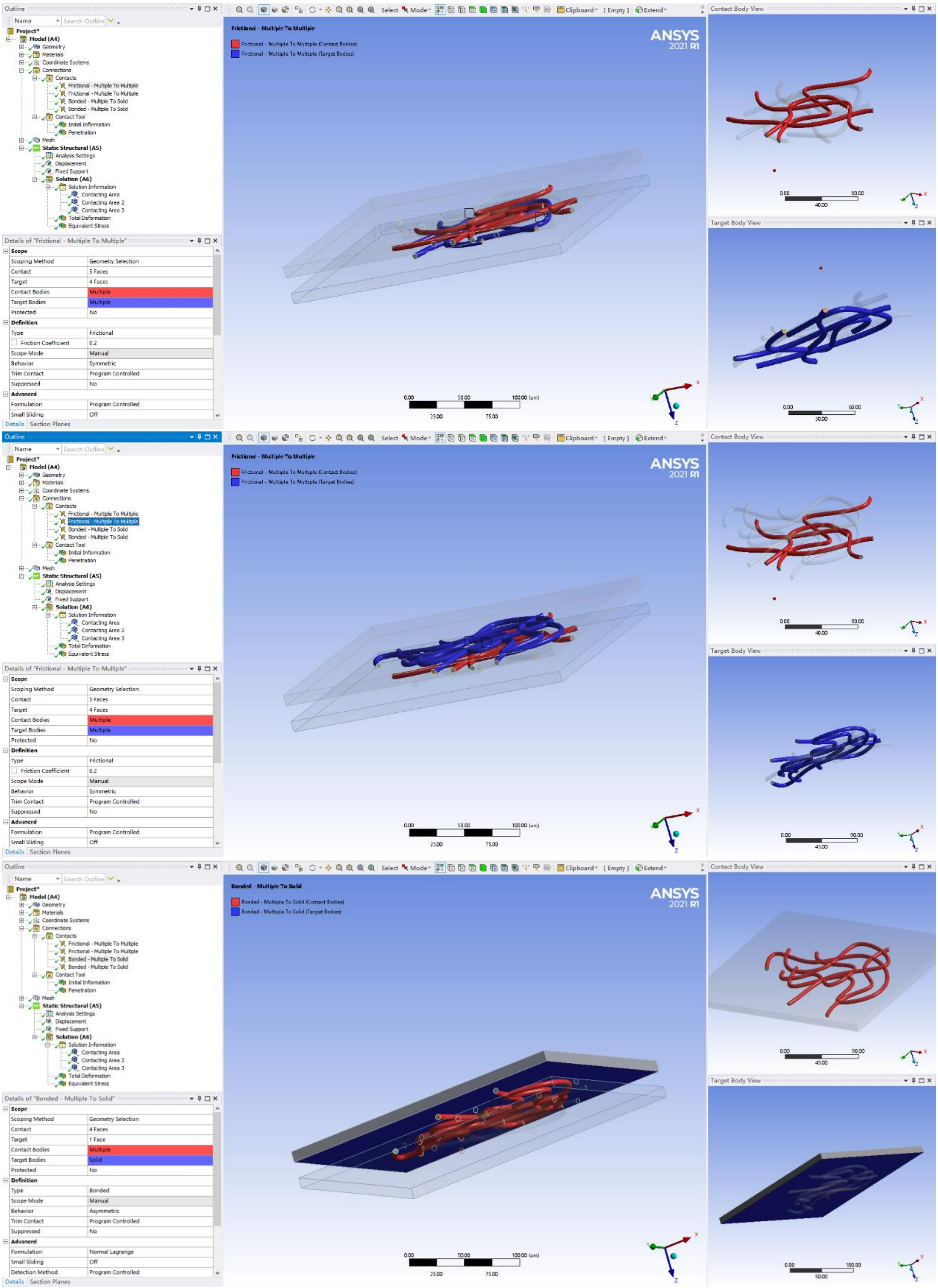
Frictional - Multiple To Multiple
Frictional - Multiple To Multiple (Contact Bodies)
Frictional - Multiple To Multiple (Target Bodies)
Bonded - Multiple To Solid
Bonded - Multiple To Solid (Contact Bodies)
Bonded - Multiple To Solid (Target Bodies)
ANSYS
2021 R1
Contact Body View
Target Body View
Outline
Details of "Frictional - Multiple To Multiple"
Details of "Bonded - Multiple To Solid"
Scope
Scoping Method
Geometry Selection
Contact
Target
Contact Bodies
Target Bodies
Multiple
Solid
Definition
Type
Frictional
Bonded
Friction Coefficient
Scope Mode
Manual
Behavior
Symmetric
Asymmetric
Trim Contact
Program Controlled
Suppressed
Advanced
Formulation
Normal Lagrange
Small Sliding
Detection Method
Static Structural (A5)
Solution (A6)
Total Deformation
Equivalent Stress

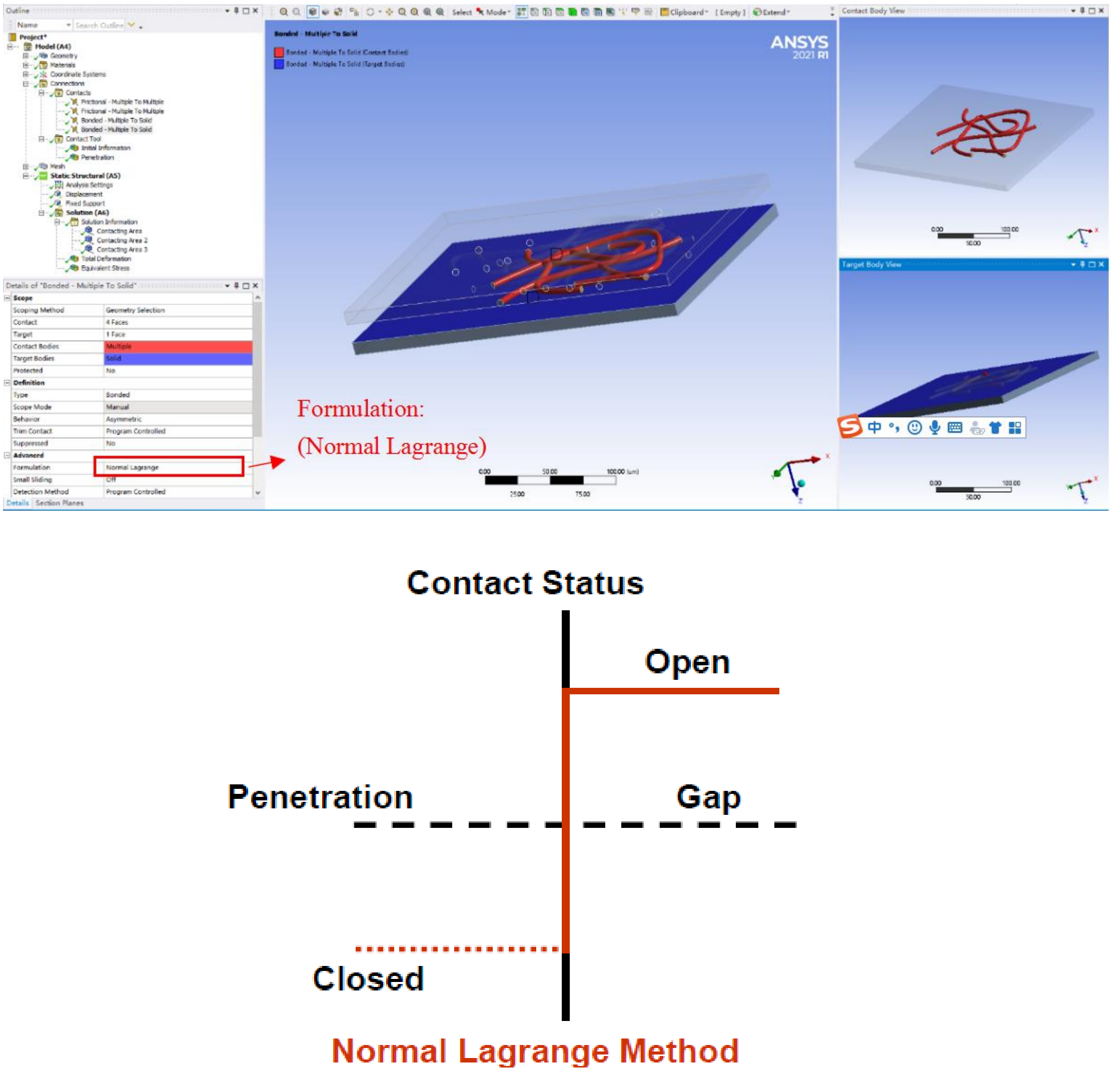

Figure S10. Contact conditions and the adopted formulation for the geometry model.

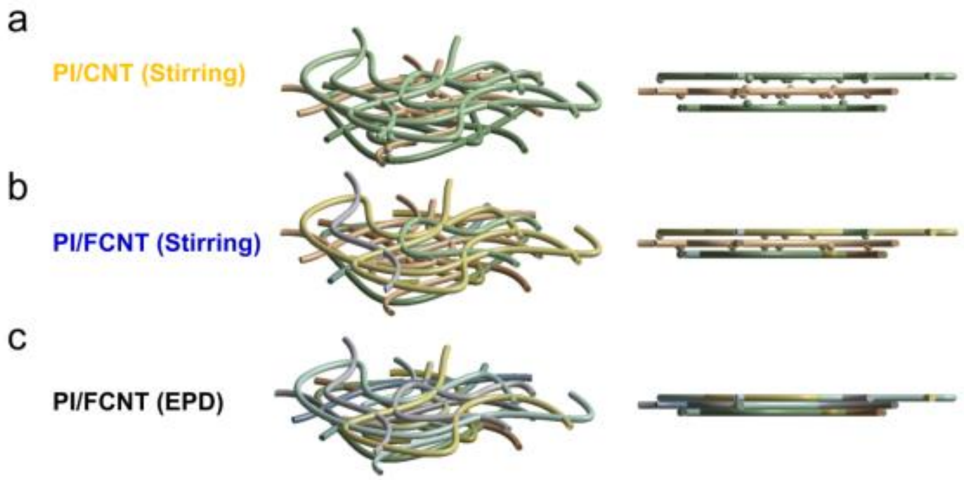

Figure S11. Established models for PI/CNT(stirring), PI/FCNT(stirring) and PI/FCNT(EPD).

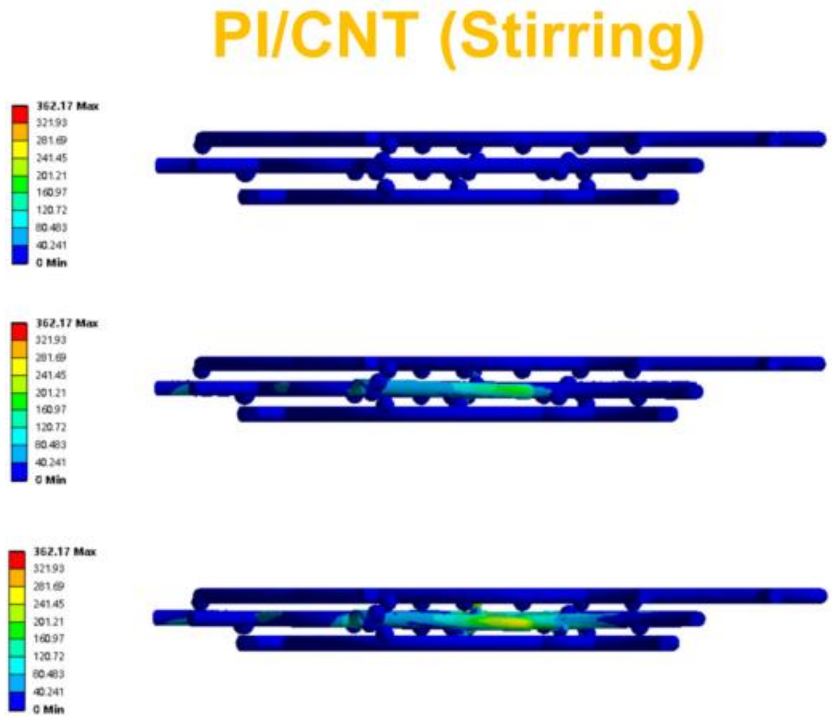

Figure S12. Dynamic microscopic deformation process for PI/CNT (Stirring) model.

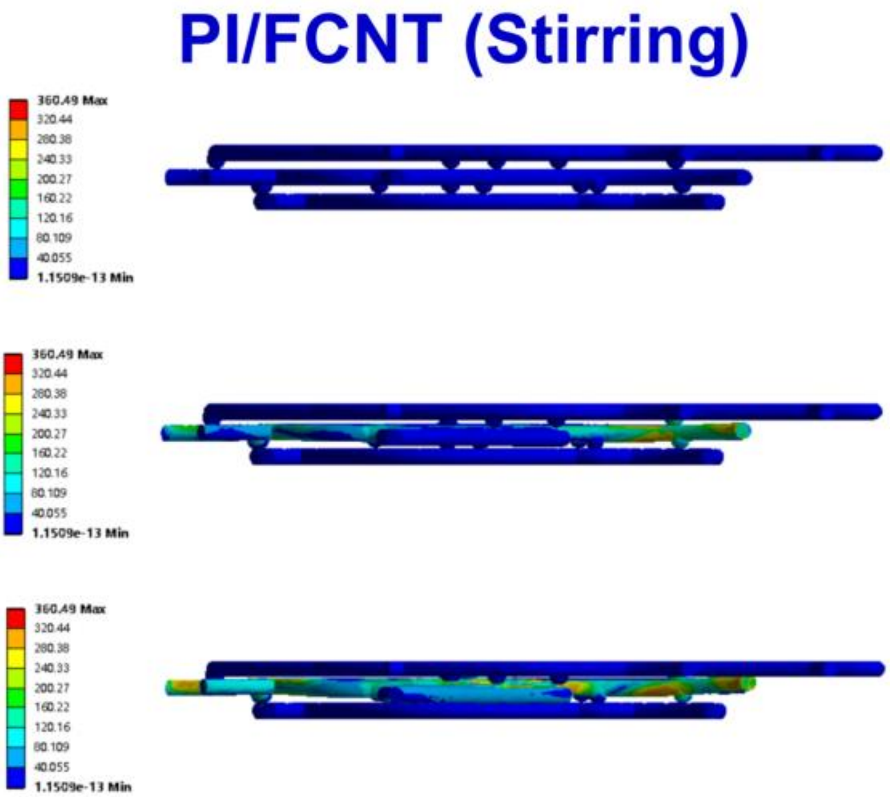

Figure S13. Dynamic microscopic deformation process for PI/FCNT (Stirring) model.

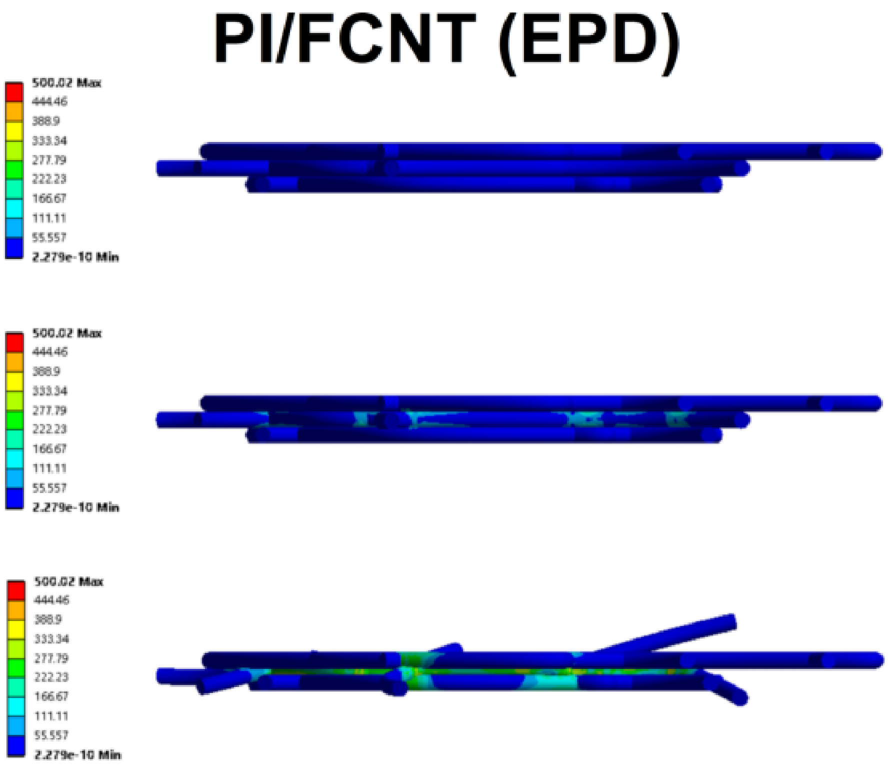

Figure S14. Dynamic microscopic deformation process for PI/FCNT (EPD) model.

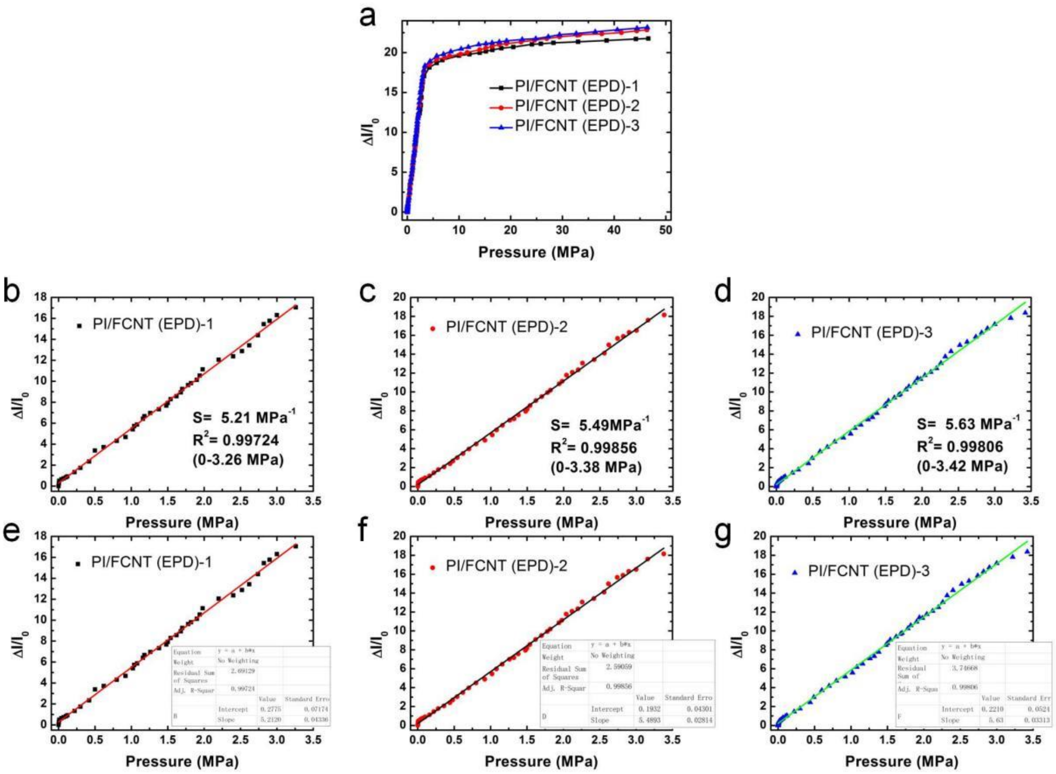

Figure S15. (a) Current response of three PI/FCNT(EPD) pressure sensors (PI fiber fabric thickness: 210 μm). (b, e) Plots of current variation versus pressures up to 3.26 MPa, and the corresponding sensitivity for PI/FCNT (EPD)-1. (c, f) Plots of current variation versus pressures up to 3.38 MPa and the corresponding sensitivity for PI/FCNT (EPD)-2. (d, g) Plots of current variation versus pressures up to 3.42 MPa and the corresponding sensitivity for PI/FCNT (EPD)-3.

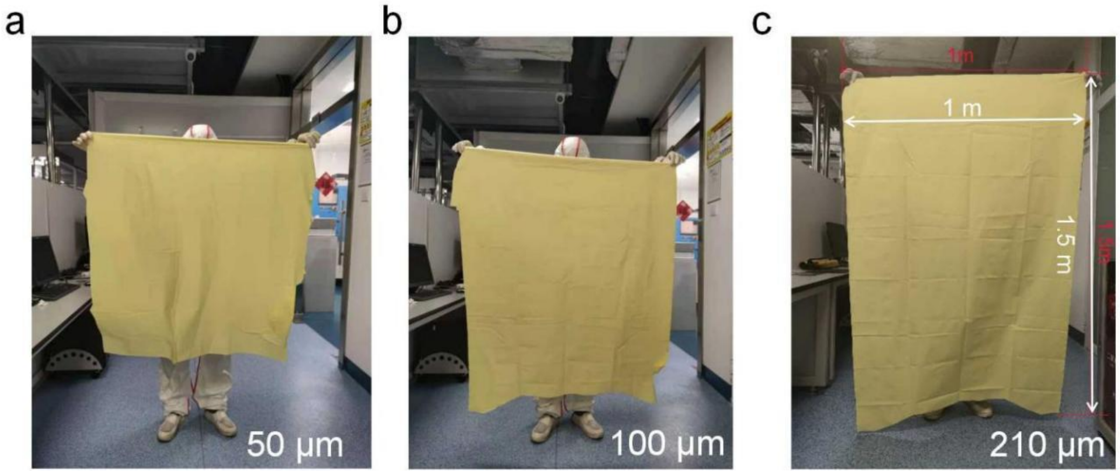

Figure S16. Fabrication of large-area PI fiber fabrics with different thicknesses. (a) 50 μm, (b) 100 μm, and (c) 210 μm.

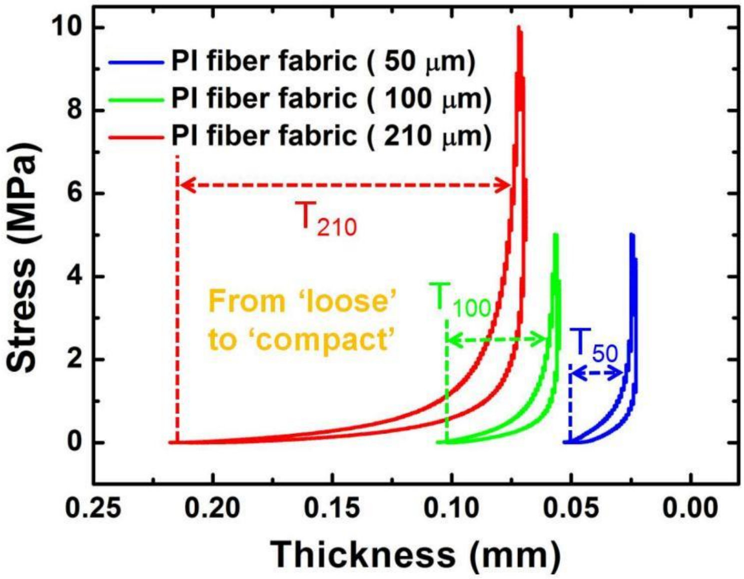

Figure S17. Compressive stress-strain curve for PI fiber fabrics with different thicknesses.

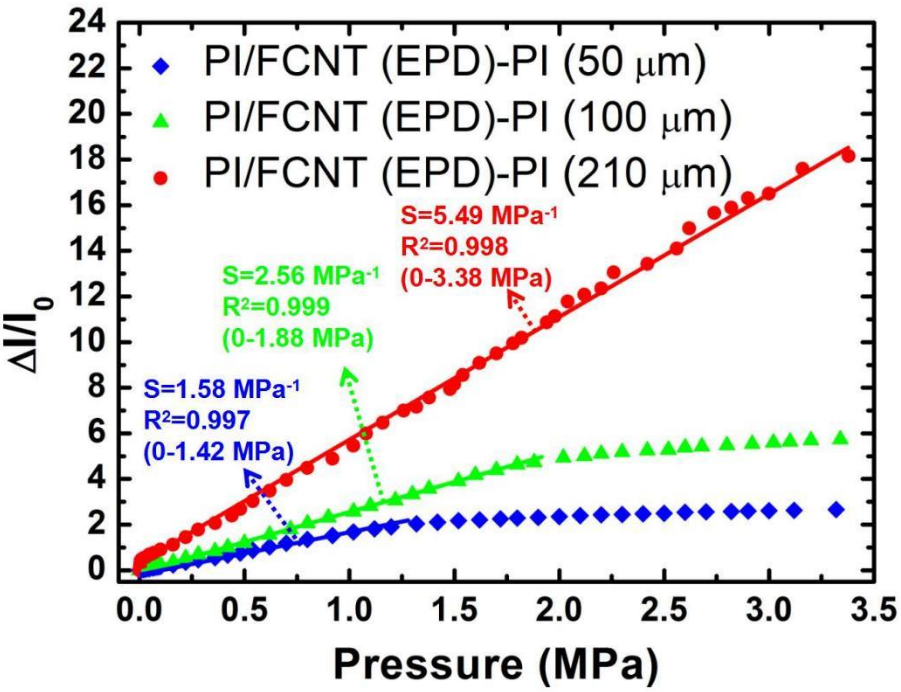

Figure S18. Relative current change *vs.* the pressure applied to the PI/FCNT(EPD) pressure sensors with the PI fiber fabric thickness of 50 μm, 100 μm and 210 μm, respectively.

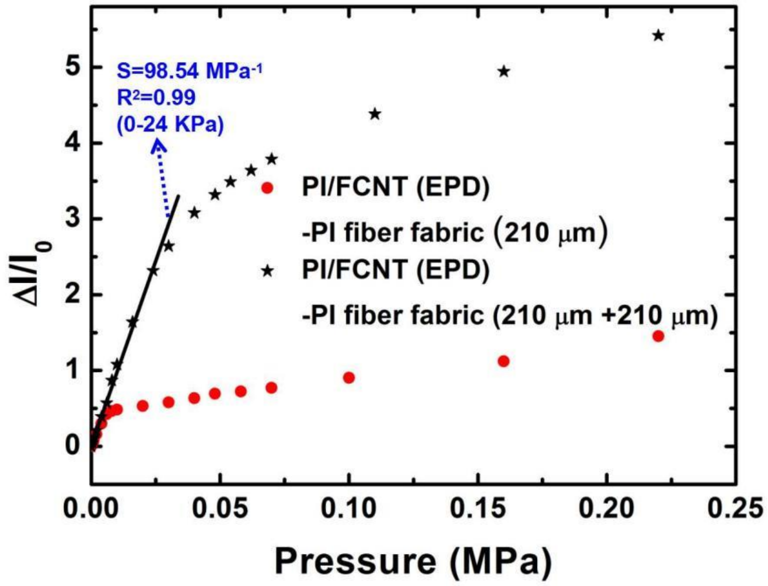

Figure S19. Relative current change *vs.* the pressure applied to the PI/FCNT (EPD) (210 μm) and PI/FCNT (EPD) (210 μm + 210 μm).

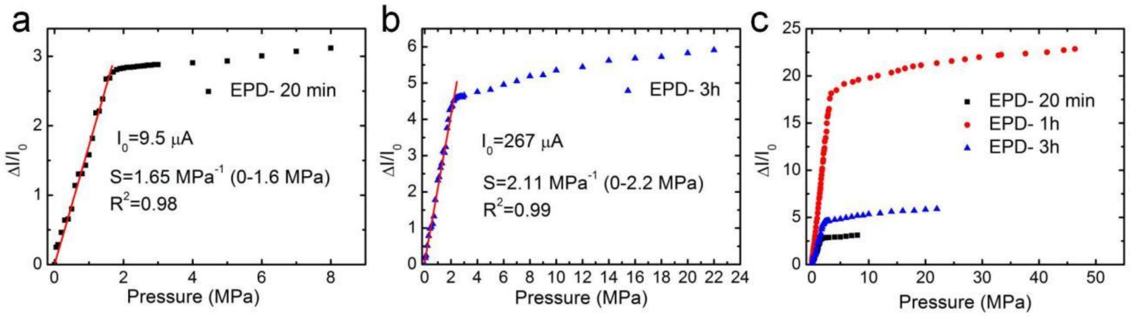

Figure S20. (a) Relative current change *vs.* the pressure applied to the PI/FCNT(EPD) pressure sensor (EPD-20 min). (b) Relative current change *vs.* the pressure applied to the PI/FCNT(EPD) pressure sensor (EPD-3 h). (c) Comparison of the P-I performance for the PI/FCNT(EPD) pressure sensors with different EPD times.

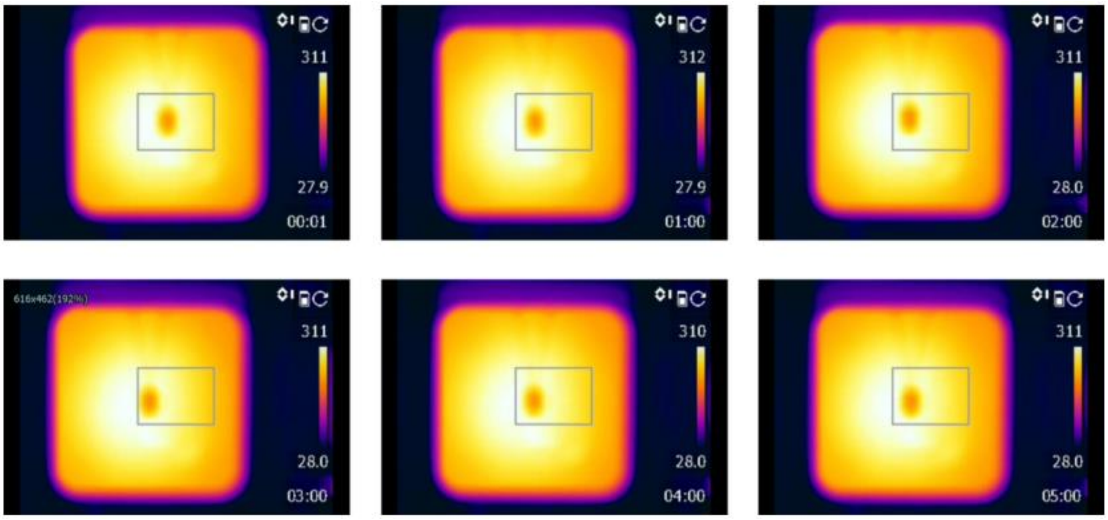

Figure S21. Infrared thermal imaging images of the PI/FCNT (EPD) pressure sensor during a heating period of 5 min.

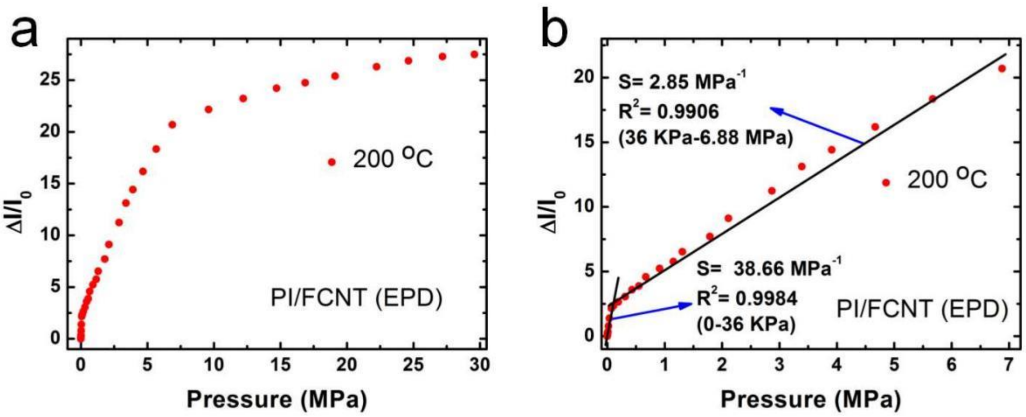

Figure S22. (a) Current response of the PI/FCNT(EPD) pressure sensors under 200 °C. (b) Plots of current variation versus pressures from 0 to 6.88 MPa.

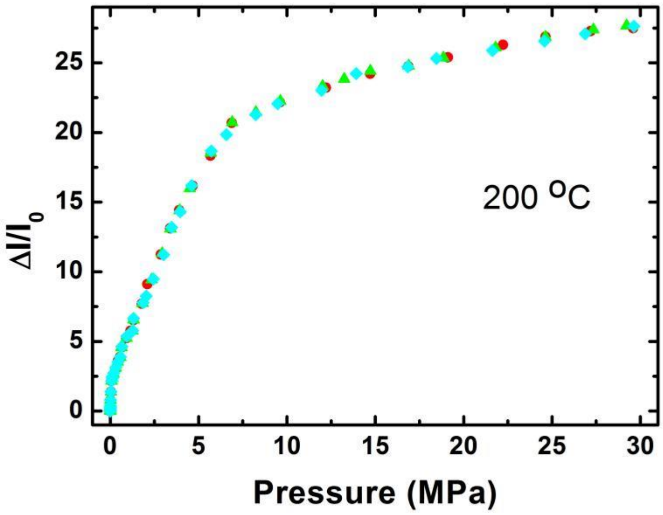

Figure S23. Current response of the PI/FCNT(EPD) pressure sensors under 200 ^{o}C (three times).

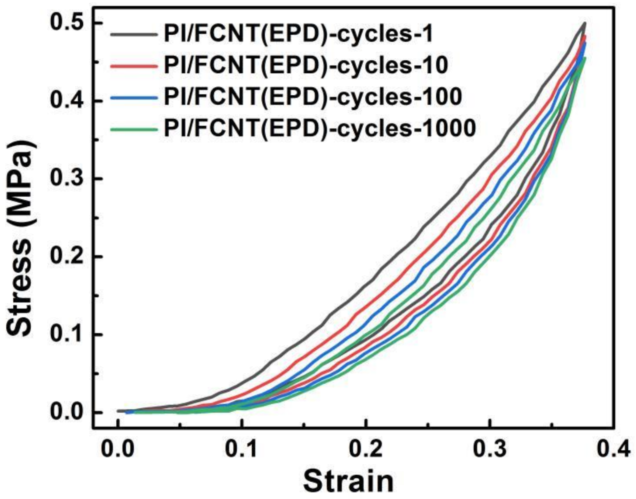

Figure S24. Cyclic compressive stress-strain curves of PI/FCNT(EPD) (room temperature).

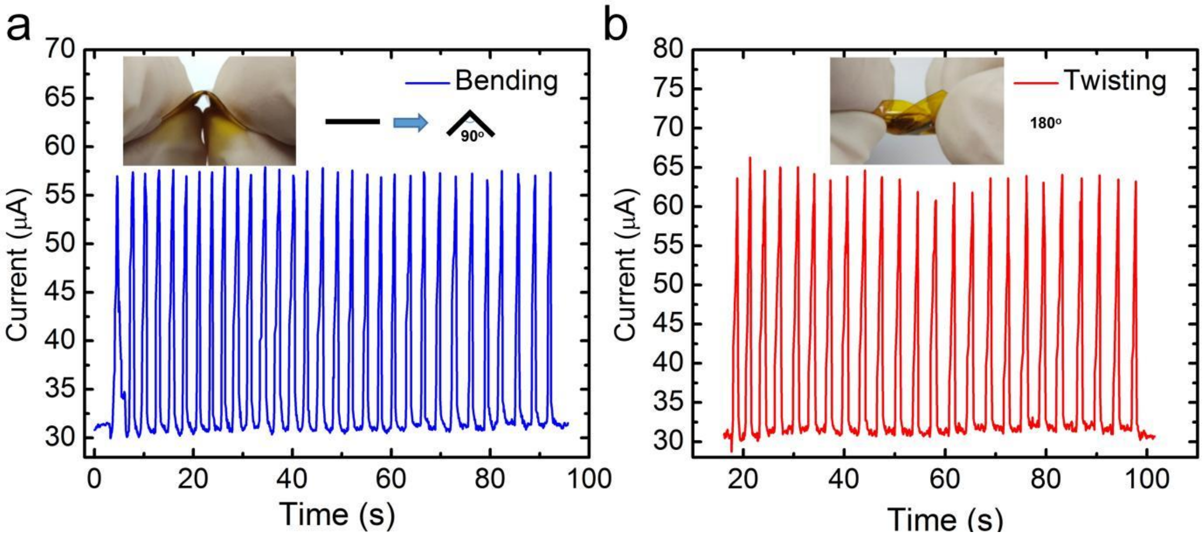

Figure S25. (a-b) Cyclic response test of the PI/FCNT(EPD) sensor device upon bending and twisting deformation, respectively.

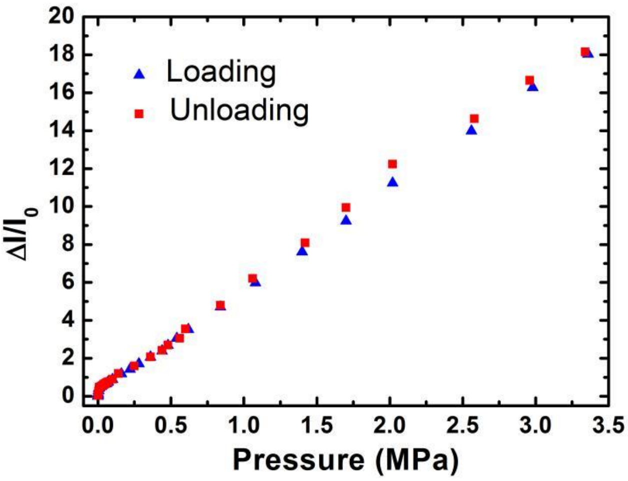

Figure S26. Electrical hysteresis of the fabricated PI/FCNT (EPD) pressure sensor obtained during its continuous loading and unloading process.

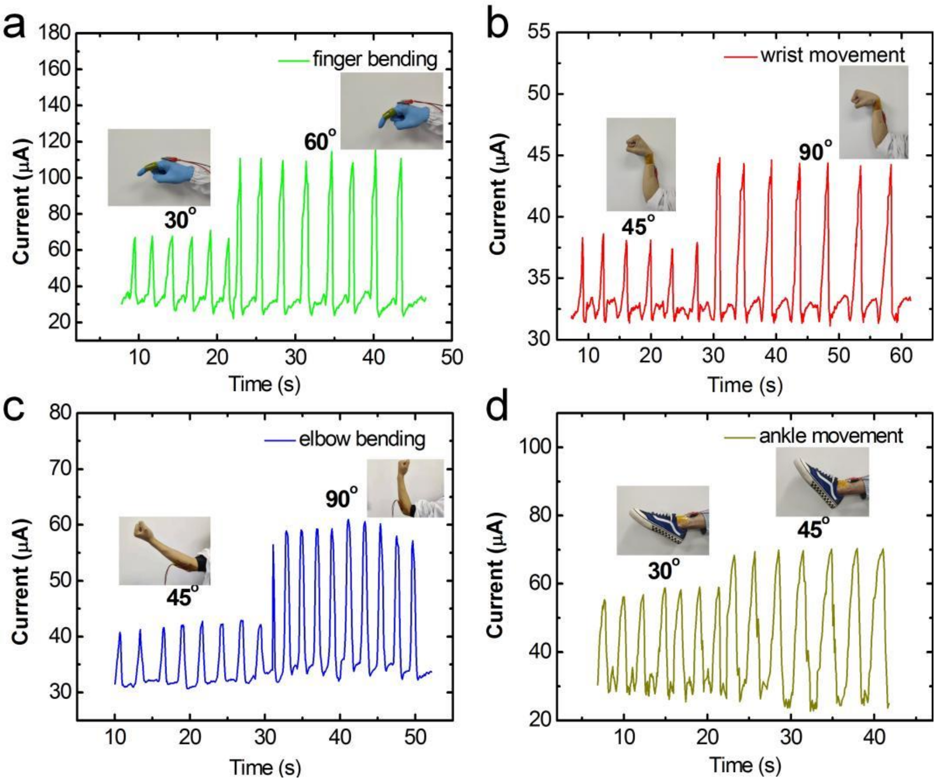

Figure S27. Detection of low pressure: monitoring finger bending, wrist movement, elbow bending, and ankle movement.

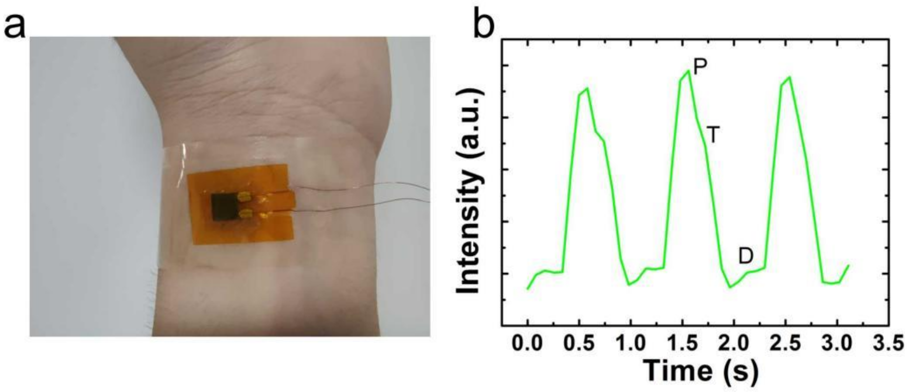

Figure S28. (a) Photograph of the PI/FCNT (EPD) pressure sensor attached to the wrist dermal area for monitoring the pulse pressure. (b) Real-time recording of the radial artery blood pressure waveform.

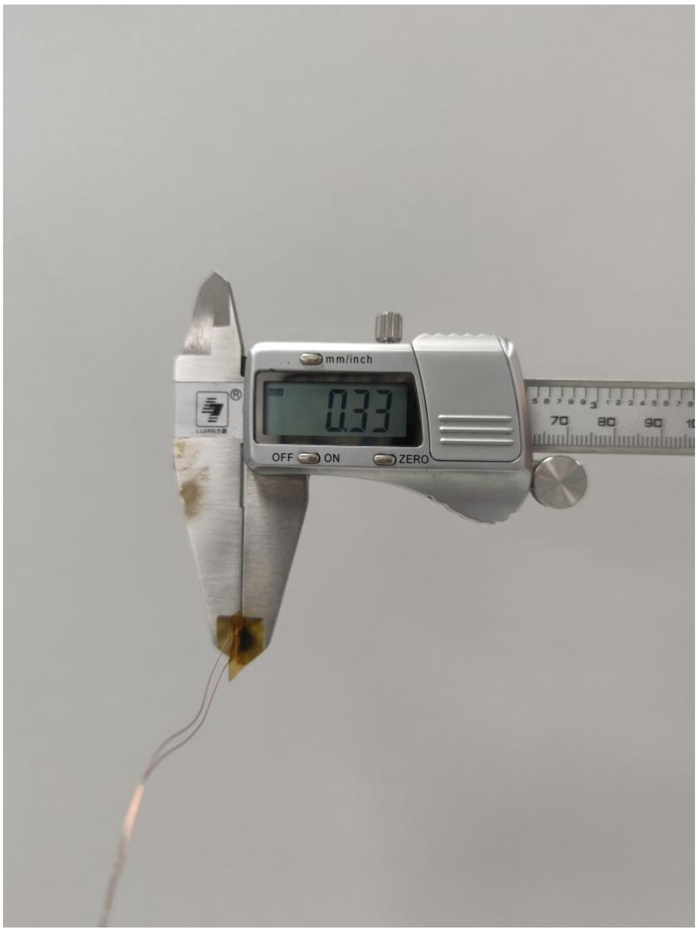

Figure S29. Optical image of the encapsulated PI/FCNT (EPD) pressure sensor. The thickness is 0.33 mm.

Table 1. Comprehensive performance comparison of the recently reported high-performance pressure sensors and our proposed PI/FCNT(EPD) pressure sensor.

References	Materials	Sensor Thickness	Sensitivity	The capability to detect the full range of faint pressure (< 100 Pa), low pressure (~ KPa) and high pressure (~ MPa)	Limit of detection	Linear Range	Total Detection Range	High temperature resistance
Nano. Lett, 2013, 13, 3237	Graphene	-	0.01 MPa^{-1}	N/A	-	-	-	-
ACS Nano 2017, 11, 4507	PI/PTNWs/Graphene	-	9.4 MPa^{-1}	N/A	-	0-1.4 KPa	≤ 1.4 KPa	-
Adv. Funct. Mater. 28 (2018), 1802576.	KCl/PVA/PAM Hydrogel	1.5 mm	50 MPa^{-1}	N/A	-	0-3.27 KPa	≤ 6.83 kPa	N/A
Compos. Sci. Technol., 2018,155 ,108-116	Graphene/silver sponge	20 mm	16 MPa^{-1}	N/A	0.28 Pa	-	0.28 Pa-50 KPa	-
Small, 2019, 15 , 1901744	PPy/PDMS	9 mm	70 MPa^{-1}	N/A	-	-	≤ 100 kPa	N/A
Chemical engineering journal, 2021, 420, 127720	PDMS/Mxene fabric/PI	>0.8 mm	5.3 KPa^{-1}	N/A	-	0-1.3 KPa	≤ 160 kPa	N/A
Adv. Mater, 2016, 28, 2601	GO/PDMS/rGO	-	2 MPa^{-1}	N/A	-	-	≤ 400 KPa	N/A
Adv. Funct. Mater., 2015, 25 , 2287 2295	Liquid metal/elastomer	0.8 mm	1 MPa^{-1}- 10 MPa^{-1}	N/A	-	-	≤ 405 kPa	N/A
ACS Appled materials &interfaces, 2016, 8, 20364	ITO/PET/Porous PDMS/ITO/PET	~1.8 mm	0.26 KPa^{-1} (0-0.33 KPa),<br><br>0.01 KPa^{-1} (0.33-250 KPa)<br><br>0.0009 KPa^{-1} (250 KPa- 1 MPa)	N/A	1 Pa	0-0.33 KPa	1 Pa-1000 KPa	N/A
Org. Electron. 2018, 53, 213.	PDMS/CNT/PEDOT:PSS	-	1.12 MPa^{-1}	N/A	-	-	≤ 1.2 MPa	N/A
This work	PI/FCNT (EPD) ---Large scale, unifrom PI fiber fabrics	~0.33 mm (encapsulated)	70.98 MPa^{-1}(0-6 KPa),<br><br>5.47 MPa^{-1}(6 KPa-3.38 MPa)<br><br>or<br><br>5.49 MPa^{-1} (0-3.38 MPa)	√	8.2 Pa (210 μm)	0-3.38 MPa (210 μm)<br><br>0-1.88 MPa (100 μm)<br><br>0-1.42 MPa (50 μm)	8.2 Pa-45 MPa (210 μm)	>350 °C
